# Urbanicity and Lifestyle Risk Factors for Cardiometabolic Diseases in Rural Uganda: A Cross-Sectional Study

**DOI:** 10.1371/journal.pmed.1001683

**Published:** 2014-07-29

**Authors:** Johanna Riha, Alex Karabarinde, Gerald Ssenyomo, Steven Allender, Gershim Asiki, Anatoli Kamali, Elizabeth H. Young, Manjinder S. Sandhu, Janet Seeley

**Affiliations:** 1 Department of Public Health and Primary Care, University of Cambridge, Cambridge, United Kingdom; 2 Wellcome Trust Sanger Institute, Hinxton, United Kingdom; 3 Medical Research Council/Uganda Virus Research Institute (MRC/UVRI) Uganda Research Unit on AIDS, Entebbe, Uganda; 4 Nuffield Department of Population Health, University of Oxford, Oxford, United Kingdom; 5 School of Health and Social Development, Deakin University, Melbourne, Australia; 6 London School of Hygiene & Tropical Medicine, London, United Kingdom; Harvard University, United States of America

## Abstract

**Background:**

Urban living is associated with unhealthy lifestyles that can increase the risk of cardiometabolic diseases. In sub-Saharan Africa (SSA), where the majority of people live in rural areas, it is still unclear if there is a corresponding increase in unhealthy lifestyles as rural areas adopt urban characteristics. This study examines the distribution of urban characteristics across rural communities in Uganda and their associations with lifestyle risk factors for chronic diseases.

**Methods and Findings:**

Using data collected in 2011, we examined cross-sectional associations between urbanicity and lifestyle risk factors in rural communities in Uganda, with 7,340 participants aged 13 y and above across 25 villages. Urbanicity was defined according to a multi-component scale, and Poisson regression models were used to examine associations between urbanicity and lifestyle risk factors by quartile of urbanicity. Despite all of the villages not having paved roads and running water, there was marked variation in levels of urbanicity across the villages, largely attributable to differences in economic activity, civil infrastructure, and availability of educational and healthcare services. In regression models, after adjustment for clustering and potential confounders including socioeconomic status, increasing urbanicity was associated with an increase in lifestyle risk factors such as physical inactivity (risk ratio [RR]: 1.19; 95% CI: 1.14, 1.24), low fruit and vegetable consumption (RR: 1.17; 95% CI: 1.10, 1.23), and high body mass index (RR: 1.48; 95% CI: 1.24, 1.77).

**Conclusions:**

This study indicates that even across rural communities in SSA, increasing urbanicity is associated with a higher prevalence of lifestyle risk factors for cardiometabolic diseases. This finding highlights the need to consider the health impact of urbanization in rural areas across SSA.

*Please see later in the article for the Editors' Summary*

## Abstract 2

Johanna Riha and colleagues evaluate the association of lifestyle risk factors with elements of urbanicity, such as having a public telephone, a primary school, or a hospital, among individuals living in rural settings in Uganda.

*Please see later in the article for the Editors' Summary*

## Editors’ Summary

**Background:**

Cardiometabolic diseases—cardiovascular diseases that affect the heart and/or the blood vessels and metabolic diseases that affect the cellular chemical reactions needed to sustain life—are a growing global health concern. In sub-Saharan Africa, for example, the prevalence (the proportion of a population that has a given disease) of adults with diabetes (a life-shortening metabolic disease that affects how the body handles sugars) is currently 3.8%. By 2030, it is estimated that the prevalence of diabetes among adults in this region will have risen to 4.6%. Similarly, in 2004, around 1.2 million deaths in sub-Saharan Africa were attributed to coronary heart disease, heart failure, stroke, and other cardiovascular diseases. By 2030, the number of deaths in this region attributable to cardiovascular disease is expected to double. Globally, cardiovascular disease and diabetes are now responsible for around 17.3 million and 1.3 million annual deaths, respectively, together accounting for about one-third of all deaths.

**Why Was This Study Done?:**

Experts believe that increased consumption of saturated fats, sugar, and salt and reduced physical activity are partly responsible for the increasing global prevalence of cardiometabolic diseases. These lifestyle changes, they suggest, are related to urbanization—urban expansion into the countryside and migration from rural to urban areas. If this is true, the prevalence of unhealthy lifestyles should increase as rural areas adopt urban characteristics. Sub-Saharan Africa is the least urbanized region in the world, with about 60% of the population living in rural areas. However, rural settlements across the subcontinent are increasingly adopting urban characteristics. It is important to know whether urbanization is affecting the health of rural residents in sub-Saharan Africa to improve estimates of the future burden of cardiometabolic diseases in the region and to provide insights into ways to limit this burden. In this cross-sectional study (an investigation that studies participants at a single time point), the researchers examine the distribution of urban characteristics across rural communities in Uganda and the association of these characteristics with lifestyle risk factors for cardiometabolic diseases.

**What Did the Researchers Do and Find?:**

For their study, the researchers used data collected in 2011 by the General Population Cohort study, a study initiated in 1989 to describe HIV infection trends among people living in 25 villages in rural southwestern Uganda that collects health-related and other information annually from its participants. The researchers quantified the “urbanicity” of the 25 villages using a multi-component scale that included information such as village size and economic activity. They then used statistical models to examine associations between urbanicity and lifestyle risk factors such as body mass index (BMI, a measure of obesity) and self-reported fruit and vegetable consumption for more than 7,000 study participants living in those villages. None of the villages had paved roads or running water. However, urbanicity varied markedly across the villages, largely because of differences in economic activity, civil infrastructure, and the availability of educational and healthcare services. Notably, increasing urbanicity was associated with an increase in lifestyle risk factors for cardiovascular diseases. So, for example, people living in villages with the highest urbanicity scores were nearly 20% more likely to be physically inactive and to eat less fruits and vegetables and nearly 50% more likely to have a high BMI than people living in villages with the lowest urbanicity scores.

**What Do These Findings Mean?:**

These findings indicate that, across rural communities in Uganda, even a small increase in urbanicity is associated with a higher prevalence of potentially modifiable lifestyle risk factors for cardiometabolic diseases. These findings suggest, therefore, that simply classifying settlements as either rural or urban may not be adequate to capture the information needed to target strategies for cardiometabolic disease management and control in rural areas as they become more urbanized. Because this study was cross-sectional, it is not possible to say how long a rural population needs to experience a more urban environment before its risk of cardiometabolic diseases increases. Longitudinal studies are needed to obtain this information. Moreover, studies of other countries in sub-Saharan Africa are needed to show that these findings are generalizable across the region. However, based on these findings, and given that more than 553 million people live in rural areas across sub-Saharan Africa, it seems likely that increasing urbanization will have a substantial impact on the future health of populations throughout sub-Saharan Africa.

**Additional Information:**

Please access these websites via the online version of this summary at http://dx.doi.org/10.1371/journal.pmed.1001683.

- This study is further discussed in a *PLOS Medicine* Perspective by Fahad Razak and Lisa Berkman
- The American Heart Association provides information on all aspects of cardiovascular disease and diabetes; its website includes personal stories about heart attacks, stroke, and diabetes
- The US Centers for Disease Control and Prevention has information on heart disease, stroke, and diabetes (in English and Spanish)
- The UK National Health Service Choices website provides information about cardiovascular disease and diabetes  (including some personal stories)
- The World Health Organization’s Global Noncommunicable Disease Network (NCDnet) aims to help low- and middle-income countries reduce illness and death caused by cardiometabolic and other non-communicable diseases
- The World Heart Federation has recently produced a report entitled “Urbanization and Cardiovascular Disease”
- Wikipedia has a page on urbanization (note that Wikipedia is a free online encyclopedia that anyone can edit; available in several languages)

## Introduction

Cardiometabolic diseases are a growing concern across sub-Saharan Africa (SSA). According to current estimates, the prevalence of diabetes among adults aged 20–79 y in Africa is 3.8% and will increase to 4.6% by 2030 [1]. Similarly, in 2004, around 1.2 million deaths were attributable to cardiovascular disease in the region, and this figure is expected to double by 2030 [2]. Urban environments and associated lifestyles, including diets high in salt, sugar, and fat, and physical inactivity, have been widely implicated as leading causes of the rise in cardiometabolic diseases [3]–[5].

Although SSA remains the least urbanized region in the world, with over 60% of the population still residing in rural areas, rural settlements across the subcontinent are increasingly adopting urban characteristics through technological improvements in transportation and telecommunication [6]–[8]. If and how these changes affect the health of rural residents, however, remains poorly understood.

Existing research on lifestyle risk factors for cardiometabolic diseases has almost exclusively focused on exposures to urban environments, using dichotomous comparisons between rural and urban populations or rural to urban migration to delineate associated risks [9],[10]. These classification schemes often rely on a single parameter to dichotomise environments and frequently categorise non-urban settlements as homogenously rural, obscuring differences in urbanicity—defined as the degree to which an area is urban—that may exist between rural environments. While multi-component scale-based definitions have been proposed as alternatives to the widely used dichotomous rural–urban classification, there is limited evidence assessing the validity of suggested metrics and associations to cardiometabolic risk factors [11]–[18]. Furthermore, all of the studies to date have investigated urbanicity across a wide spectrum of settlements that included at least one urban centre; none of the studies have examined associations solely within rural settlements [11]–[16]. Therefore, it is impossible to comment on whether there is an association between an increased presence of urban characteristics and cardiometabolic risk in rural populations. It is also worth noting that almost all of the multi-component scale-based studies were set in Asia, making it difficult to generalise findings outside of the Asian continent [11]–[16].

Understanding how lifestyle risk factors for cardiometabolic diseases accrue as rural environments across SSA become more urbanized is important for multiple reasons; such understanding would provide (1) important information on the natural progression of population health states through different stages of urbanization, (2) a better indication of future burdens of cardiometabolic diseases, and, importantly, (3) insights into potential avenues for intervention given that lifestyle risk factors for cardiovascular diseases are potentially modifiable. Considering the lack of data on the health effects of urbanization in rural areas and the estimate that more than 533 million people live in rural areas across SSA, we completed a cross-sectional study assessing variations in urbanicity levels across 25 rural Ugandan settlements and whether lifestyle risk factors were associated with increasing urbanicity across these communities [19].

## Methods

Ethical approval for the study was obtained from the Science and Ethics Committee of the Uganda Virus Research Institute (UVRI), the Ugandan National Council for Science and Technology, and the East of England–Cambridge South (formerly Cambridgeshire 4) National Health Services Research Ethics Committee, UK.

### Study Population

**Figure 1:**
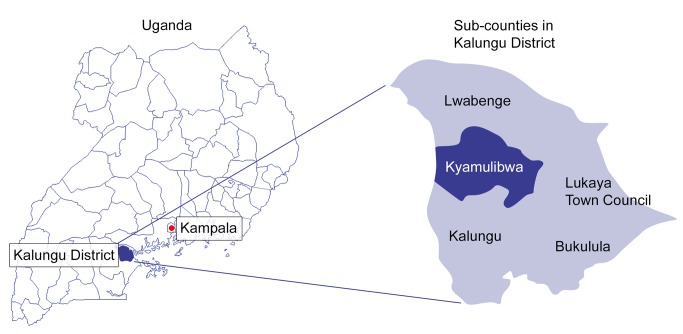
Map of districts within Uganda and sub-counties within Kalungu District.

This study was set in Kyamulibwa sub-county of Kalungu district in rural southwestern Uganda, shown in Figure 1. The sub-county is located in the central region of Uganda, about 130 km southwest of the capital city, Kampala [20]. The land area of Kyamulibwa is 56.3 km^2^, with a total population over 33,000 [20]. The smallest administrative unit is a “village”, with boundaries delineated by the government; there are 53 villages in the sub-county, varying in size from 300 to 1,500 residents [20]. There are no paved roads throughout the sub-county, and no households have running water. At the centre of the study area is a research field station belonging to the Medical Research Council/Uganda Virus Research Institute (MRC/UVRI), which includes a public medical clinic, administration offices, laboratory, and its own water supply system [21].

Our study was nested within an existing cohort, the General Population Cohort (GPC). The design and methods of the cohort have been published elsewhere [21]. In brief, the GPC is a population-based cohort of around 25,000 people living within a cluster of 25 villages in Kyamulibwa. The cohort was established in 1989 by the MRC/UVRI Programme on AIDS to describe trends in the prevalence and incidence of HIV infection and their determinants in the general population. At the time of the study, the study population was assessed through annual house-to-house “rounds” of both a census and a survey, during which demographic, socio-medical, and serological data were collected. Other health-related information was also gathered, with topics varying annually. All study participants gave informed consent for each census and survey round prior to enrolment. This study uses census and survey data from round 22 of the GPC, completed during 2011. The sampling frame for the census was all households within Kyamulibwa sub-county with at least one adult or emancipated minor who had spent or was planning to spend at least 3 mo in the household [21]. All residents aged 13 y and above were eligible for the survey. Over 95% of households approached for the census participated, and over 80% of eligible census participants took part in the survey. Although we do not have information on households and individuals who did not participate in round 22 of the GPC, those included in round 22 are likely to be representative of residents in Kyamulibwa, given the high proportion who participated in the study (>80%). Round 22 of the GPC included census data from 3,771 households and medical survey data from 7,830 residents aged 13 y and above [21].

### Urbanicity Score

Following a review of literature, we selected an existing multi-component urbanicity scale based on the following criteria: (1) content validity and reliability, (2) validation of the scale in multiple low- and middle-income countries (LMICs), and (3) availability of data and ease of implementation within the GPC area. A scale developed by Novak et al., previously validated in Ethiopia, Peru, and India, was used to quantify how urban each of the 25 villages in Kyamulibwa was [14]. The scale’s scoring system was based on seven components—population size, economic activity, built environment, communication services, educational facilities, health services, and diversity, which comprised two separate scores related to variance in housing quality and variance in the number of years women have spent in education [14]. The urbanicity scale used in our analyses differed to the one used by Novak et al. in two minor ways. First, because relevant data were not available, we were unable to attribute a score (4 points) for the proportion of households with a television or a mobile phone in the “communication services” component of the urbanicity scale. Second, we did not include a score for housing quality (5 points) in the “diversity” component of the urbanicity score. Instead, we considered housing quality to be a marker for socioeconomic status (SES), a potential confounder in our analyses (see “Covariates” below). Literature suggests that both asset ownership and housing quality are indicators of SES and are strongly associated with health outcomes [22]–[26]. These two changes meant our maximum possible urbanicity score was 61 points, with a possible range from 61 points (very urban) to 0 points (very rural), compared to the 70-point scale used by Novak et al. [14]. The scoring algorithm used in our study is shown in Table 1.

**Table 1:** Scoring algorithm used for the urbanicity scale.

Component	Score Item	Scale Scoring
**Population size**	**Approximate number of people (including children) living in the locality**	
	1–500	1 point
	501–1,000	2 points
	1,001–2,000	3 points
	2,001–4,000	4 points
	4,001–6,000	5 points
	6,001–8,000	6 points
	8,001–10,000	7 points
	10,001–15,000	8 points
	15,001–20,000	9 points
	>20,000	10 points
**Economic activity**	**Proportion of the population involved in agriculture (primary occupation)** †	10 points – 10 × proportion of population involved in agriculture†
**Built environment**	**Types of roads in locality**	
	Paved roads	2 points
	Unpaved roads for motor traffic	1 point
	Non-motorised roads	0 points
	**Sewage services**	
	Sewage system in locality	2 points
	Proportion of households with a flush toilet	2 points × proportion of households with a flush toilet
	**Electricity services**	
	Electricity in locality	2 points
	Proportion of households with electricity	2 points × proportion of households with electricity
**Communication services**	**Communication services in locality**	
	Movie theatre	2 points
	Public internet	2 points
	Public telephone	2 points
**Education facilities**	**Educational facilities in locality**	
	Nursery and/or preschool	2 points
	Primary school	2 points
	Secondary school	2 points
	University	2 points
	**Average education of women in the locality**	Average number of years of education/6∧
**Health services**	**Health facilities in locality**	
	Hospital (public or private)	2 points
	Health centre (public or private)	2 points
	Dispensary/pharmacy	2 points
	**Health workers available in locality**	
	Midwife	2 points
	Health worker	2 points
**Diversity**	**Variance in women’s education**	
	Decile 9	4.5 points
	Decile 8	4 points
	Decile 7	3.5 points
	Decile 6	3 points
	Decile 5	2.5 points
	Decile 4	2 points
	Decile 3	1.5 points
	Decile 2	1 point
	Decile 1	0.5 points

† Proportion of the population refers to the proportion of the adult population (those aged ≥18 y).

∧The average number of years of education has been divided by six so that the total score for the “Education facilities” component is no more than 10 points.

**Figure 2:**
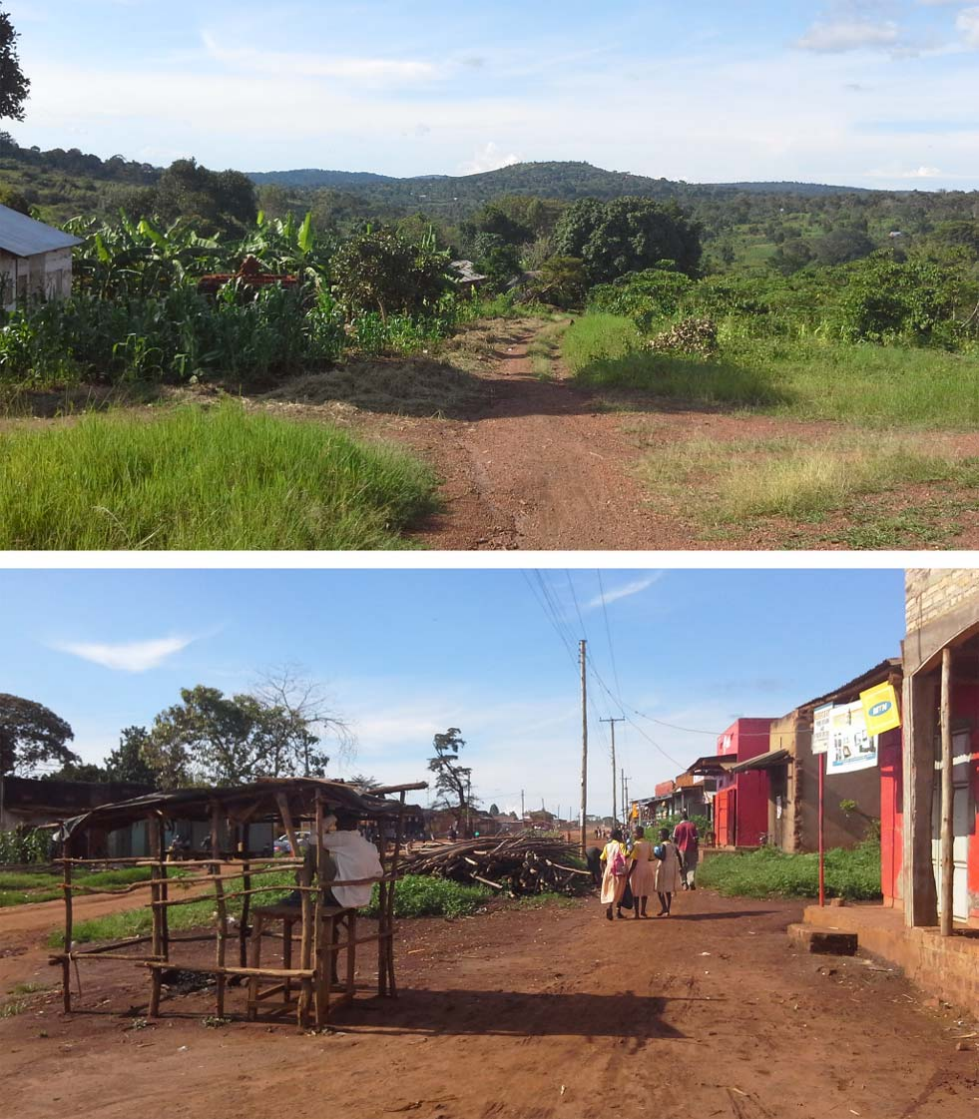
The main road in study villages in the lowest and highest urbanicity quartiles, Kyamulibwa, Uganda, 2011. (A) Village in the lowest urbanicity quartile; (B) village in the highest urbanicity quartile.

Since there is no gold standard measure for urbanicity and the sub-county of Kyamulibwa is classified as homogenously rural by the Ugandan government, we assessed the face validity of our urbanicity scale using methods consistent with a previous study [12]. This included visiting and comparing the main streets of study villages as well as asking local staff in Kyamulibwa to review the urbanicity scores based on their knowledge of the study villages. Face validity assessments confirm that our urbanicity scores captured a range in urbanicity across study villages. Figure 2 illustrates the differences in the built environment observed between study villages in the lowest and highest urbanicity quartiles.

Data used to calculate the urbanicity score were collected from multiple sources, namely, the census and survey in round 22 of the GPC, the sole electricity supplier in the sub-county, and interviews with local MRC/UVRI field staff who have worked in Kyamulibwa for over 10 y. Most of these data were from existing sources, apart from the interviews with MRC/UVRI staff, which were collected specifically for this study. All data sources used the same government-defined boundaries for each village, and all data collection took place in 2011, the same year that individual-level demographic and lifestyle information was obtained during round 22 of the GPC. Urbanicity scores were calculated for each of the 25 villages based on standardised village boundaries delineated by the government. Table 2 gives a breakdown by village of all the data items used to compute the urbanicity score.

**Table 2:** Data used to calculate urbanicity scores for each village in Kyamulibwa.

Village Number	Population Size—Total†	Proportion of Population Involved in Agriculture◊	Type of Roads in Locality (Both Motorised and Non-Motorised)*	Sewage Services in Locality*	Proportion of Households with a Flush Toilet*	Electricity in Locality▴	Proportion of Households with Electricity▴	Movie Theatre in Locality*	Public Internet in Locality*	Public Telephone in Locality*	Nursery/Preschool in Locality*	Primary School in Locality*	Secondary School in Locality*	University in Locality*	Average Number of Years of Education of Women in Locality◊	Variance in Women’s Education (Decile)◊	Hospital in Locality*	Health Centre in Locality*	Dispensary in Locality*	Local Drug Shop in Locality*	Midwifes in Locality*	Health Workers in Locality*	Bars in Locality*
1	736	0.778	Unpaved roads	None	None	No	0	No	No	Yes	No	No	No	No	7.33	8	No	No	No	No	Yes	Yes	Yes
2	347	0.815	Unpaved roads	None	None	No	0	No	No	No	No	Yes	No	No	7.36	8	No	No	No	No	Yes	Yes	No
3	904	0.806	Unpaved roads	None	None	No	0	No	No	No	No	No	No	No	6.74	6	No	No	No	No	Yes	Yes	Yes
4	663	0.687	Unpaved roads	None	None	No	0	No	No	No	No	No	No	No	7.83	9	No	No	No	No	Yes	Yes	Yes
5	965	0.849	Unpaved roads	None	None	No	0	No	No	No	No	No	No	No	6.40	5	No	No	No	No	Yes	Yes	Yes
6	1,089	0.272	Unpaved roads	None	None	Yes	0.136	Yes	No	Yes	Yes	No	Yes	No	10.03	10	No	No	Yes	Yes	Yes	Yes	Yes
7	585	0.558	Unpaved roads	None	None	Yes	0.147	No	No	No	Yes	No	No	No	9.05	10	No	No	No	No	Yes	Yes	Yes
8	683	0.754	Unpaved roads	None	None	No	0	No	No	No	No	No	No	No	6.99	7	No	No	No	No	Yes	Yes	Yes
9	1,131	0.806	Unpaved roads	None	None	No	0	No	No	No	No	Yes	No	No	5.80	2	No	No	No	No	Yes	Yes	Yes
10	564	0.692	Unpaved roads	None	None	Yes	0.054	No	No	No	No	No	Yes	No	6.79	7	No	No	No	No	Yes	Yes	Yes
11	725	0.683	Unpaved roads	None	None	No	0	No	No	No	No	No	No	No	6.04	4	No	No	No	No	Yes	Yes	Yes
12	870	0.839	Unpaved roads	None	None	No	0	No	No	No	No	Yes	No	No	5.84	2	No	No	No	No	Yes	Yes	Yes
13	512	0.860	Unpaved roads	None	None	No	0	No	No	No	No	No	No	No	5.61	1	No	No	No	No	Yes	Yes	Yes
14	650	0.855	Unpaved roads	None	None	No	0	No	No	No	No	Yes	No	No	7.16	7	No	No	No	No	Yes	Yes	Yes
15	877	0.797	Unpaved roads	None	None	No	0	No	No	No	No	No	No	No	5.84	2	No	No	No	No	Yes	Yes	Yes
16	807	0.740	Unpaved roads	None	None	Yes	0.047	No	No	No	No	Yes	No	No	7.40	8	No	No	No	No	Yes	Yes	Yes
17	1,690	0.538	Unpaved roads	None	None	Yes	0.086	No	No	Yes	No	Yes	Yes	No	8.37	9	No	No	Yes	No	Yes	Yes	Yes
18	905	0.780	Unpaved roads	None	None	No	0	No	No	No	No	No	No	No	5.17	1	No	No	No	No	Yes	Yes	Yes
19	788	0.809	Unpaved roads	None	None	No	0	No	No	No	No	Yes	No	No	5.68	2	No	No	No	No	Yes	Yes	Yes
20	1,162	0.812	Unpaved roads	None	None	No	0	No	No	No	No	Yes	No	No	5.90	3	No	No	No	No	Yes	Yes	Yes
21	1,052	0.779	Unpaved roads	None	None	No	0	No	No	No	No	No	No	No	6.00	4	No	No	No	No	Yes	Yes	Yes
22	622	0.819	Unpaved roads	None	None	No	0	No	No	No	No	No	No	No	4.87	1	No	No	No	No	Yes	Yes	Yes
23	934	0.772	Unpaved roads	None	None	No	0	No	No	No	No	Yes	No	No	6.38	5	No	No	No	No	Yes	Yes	No
24	941	0.749	Unpaved roads	None	None	No	0	No	No	No	No	Yes	No	No	6.66	6	No	No	No	No	Yes	Yes	Yes
25	843	0.782	Unpaved roads	None	None	No	0	No	No	No	No	No	No	No	6.14	5	No	No	No	No	Yes	Yes	Yes

† Data from GPC round 22 census.

◊ Data from GPC round 22 survey—refers to the adult population (those aged ≥18 y).

^*^Data from MRC/UVRI field staff interviews.

▴ Data from local electricity supplier.

Data on all components of the urbanicity score, apart from primary occupation, which was used to compute the economic activity score, were complete. Since the proportion of the population involved in agriculture was calculated based on those aged ≥18 y, proportions of adults with missing data were calculated by urbanicity quartile. We then assessed differences in demographic characteristics and risk factor data between adults with and without primary occupation data overall and by urbanicity quartile.

We used principal component factoring and oblique rotation in our factor analysis to test whether the seven components of the scale measured a single latent construct, namely, urbanicity. We also compared other approaches including principal axis factoring, which gave similar eigenvalues and numbers of factors. Two factors had an eigenvalue greater than 1 (4.4 for the first factor and 1.1 for the second); all subsequent factors had eigenvalues less than 0.6. A Cronbach’s α reliability coefficient of 0.86 also indicated that the internal consistency of the scale was good.

### Outcomes

Seven widely accepted lifestyle risk factors were selected for our analyses based on availability of data from round 22 of the GPC. The risk factors selected were current smoking, heavy drinking, low fruit and vegetable consumption, low physical activity, high body mass index (BMI), high waist circumference (WC), and high blood pressure (BP). An adaptation of the World Health Organization STEPwise Approach to Surveillance questionnaire was used to collect data on the seven outcomes [27]. Smoking, alcohol consumption, fruit and vegetable consumption, and physical activity were based on self-reported measures. Height and weight were measured using a Seca Leicester stadiometer to the nearest 0.1 cm and a Seca 761 mechanical scale to the nearest 1 kg, respectively. BMI was calculated as weight (kg)/height (m)^2^. WC was measured twice over one layer of light clothing using the Seca 201 Ergonomic Circumference Measuring Tape to the nearest 0.1 cm. A third measurement was taken if the first two measurements differed by more than 3 cm. WC was then calculated as the mean of the two (or three, where applicable) measurements. Women in their second or third trimester of pregnancy were excluded from physical measurements. BP was measured using an Omron M4-I BP monitor. To ensure use of proper-sized cuffs, arm circumference measurements were taken. Three BP readings were taken 5 min apart for each participant while they were seated. The mean of the second and third readings was taken as BP.

For all primary analyses, outcomes were reclassified as binary variables with lifestyle risk factors defined as follows: current smoking was defined as any participant who was a current smoker; heavy drinking was defined as women who reported drinking more than one drink per day or men who reported drinking more than two drinks per day; low fruit and vegetable consumption was defined as eating less than five portions of fruit or vegetables per day; low physical activity was defined as doing less than 5 d a week of any combination of walking or moderate- or vigorous-intensity activities and less than 600 min of physical activity per week; high BMI was defined as BMI ≥25 kg/m^2^; high WC was defined as WC≥94 cm for men and WC≥80 cm for women; and high BP was defined as systolic BP≥140 mm Hg or diastolic BP≥90 mm Hg or reported treatment for high BP [28]–[31]. The cutoff values for outcome variables were selected based on current World Health Organization recommendations for healthy living (fruit and vegetable consumption and physical activity), recommendations by the International Diabetes Federation on WC cutoff values for sub-Saharan Africans (WC), or established clinical guidelines for the prevention of hypertension (smoking, drinking, BMI, and BP) [32]–[36].

### Covariates

Age and SES were considered as potential confounders of the association between urbanicity and lifestyle risk factors [37]. Principal component analysis (PCA) was used to construct a variable capturing SES at the household level based on data collected from round 22 of the GPC [38]. A household-level, instead of an individual-level, variable of SES was chosen for several reasons. First, goods and services, which are related to wealth and family income, are frequently shared among household members and are often indicative of the standard of living and life chances among household members [22]. Second, economic indicators of SES related to wealth have been reported to be most strongly associated with health outcomes when compared to education and occupation, which are individual-based dimensions [22],[23]; this is particularly true for women because women tend to receive lower income returns than men from equivalent education [22],[23]. And third, it is difficult to differentiate household-level from individual-level indicators of SES. Six household variables were included in the PCA—roof material type, roof quality, wall material type, ratio of number of rooms in a house to number of people living in that household, ownership of house and land, and employment of workers for household or land. All individuals from the same household were assigned the same SES value. The first component of the PCA output was taken to be the continuous SES variable. Education and occupation of the head of household were not included in the PCA as these variables are reported to be correlated with urban settlement and were used to generate the urbanicity scale [9],[39].

### Statistical Analyses

As the aim of our study was to investigate whether increasing levels of urbanicity in rural settings were associated with an increase in unhealthy lifestyle factors, urbanicity was divided into quartiles. Each quartile was coded as its respective midpoint value since urbanicity scores across the 25 villages were not normally distributed. Analyses were restricted to individuals with data on age, sex, SES, and at least one lifestyle risk factor. Prevalence was calculated for each binary outcome (lifestyle risk factor), stratified by sex and quartile of urbanicity. Since there were clear differences in the prevalence of some lifestyle risk factors between men and women, all subsequent analyses were run for the total study population as well as stratified by sex. Since most outcomes were common (prevalence≥10%), we used Poisson regression with robust standard errors to explore crude and adjusted associations, accounting for the effects of potential confounders, such as age and SES. Fully adjusted models were multilevel mixed-effects Poisson regression models with robust standard errors that included random effects, adjusting for clustering at household level in addition to potential confounders. A random intercept for household was added to our mixed-effects models to adjust for clustering at household level. Villages in the lowest urbanicity (least urban) quartile were treated as the reference category.

Sensitivity analyses explored associations considering outcomes as continuous variables (linear regression), the exposure as a continuous variable (Poisson regression), as well as both exposure and outcomes as continuous variables (linear regression models). Confidence intervals (CIs) in all models were based on sandwich estimates of variance. Models with urbanicity as a continuous variable were based on associations relating to a 1–standard deviation (SD) change in urbanicity in order to facilitate interpretation of results. Data on number of cigarettes smoked were not available, and therefore we could not assess smoking as a continuous variable. To assess non-linearity, we ran several likelihood ratio tests considering outcomes as binary and continuous data, comparing models with urbanicity as a categorical and as a continuous variable, with the former model nested in the latter. Where there was suggestive evidence of non-linearity, we ran fractional polynomial models with continuous variables for urbanicity and each outcome to explore the shape of these associations further. We also ran post hoc exploratory analyses to test whether there was statistical evidence of interaction between sex and urbanicity on associations with lifestyle risk factors in the final model from our primary analyses.

Crude and corresponding fully adjusted models in all analyses were all restricted to include individuals with complete data (i.e., all crude and corresponding fully adjusted models were based on the same sample size). Proportions of participants with missing data for each variable were calculated by urbanicity quartile. Since the majority of participants were excluded because of missing SES data, we assessed differences in demographic characteristics and risk factor data between individuals with complete and incomplete SES data. In addition we re-ran fully adjusted Poisson and linear regression models considering urbanicity scores re-calculated based on assumptions that either all or none of the adults with missing primary occupation data were involved in agriculture as their primary occupation. All analyses were performed using STATA version 11 (StataCorp).

## Results

### Levels of Urbanicity in Kyamulibwa

Despite villages being perceived as homogenously rural, there was a clear gradient in urbanicity across the 25 villages in the GPC study area, with urbanicity scores ranging from 8 (very rural) to 32 (more urban). Table 3 shows the variation in urbanicity scores across the 25 villages. Economic activity, education facilities, communication services, and diversity contributed the most to the variation in urbanicity scores observed. In the bottom 80.0% (95% CI: 58.7%, 92.4%) of villages for urbanicity score, there were no educational facilities and no households with electricity, and the average number of years that women spent in education was less than 6 y. In the top 20.0% (95% CI: 7.6%, 41.3%) of villages for urbanicity score, up to 14.8% of households had electricity; there was at least one nursery, primary, or secondary school in the village; and women on average spent at least 6 y in education. The two most urban villages (villages 6 and 17) also had a public telephone and a dispensary, and less than 54% of inhabitants were involved in agriculture as their primary occupation. None of the villages had a paved road or sewage services, but all of the villages had a healthcare worker and a midwife.

**Table 3:** Urbanicity scores by component and village, General Population Cohort, Uganda, 2011.

Village Number	Points by Component							Total Points	Urbanicity Quartile
	Population Size	Economic Activity	Built Environment	Communication Services	Education Facilities	Health Services	Diversity		
13	2.00	1.40	1.00	0.00	0.94	2.00	0.50	7.84	1
22	2.00	1.81	1.00	0.00	0.81	2.00	0.50	8.12	1
18	2.00	2.20	1.00	0.00	0.86	2.00	0.50	8.56	1
15	2.00	2.03	1.00	0.00	0.97	2.00	1.00	9.00	1
5	2.00	1.51	1.00	0.00	1.07	2.00	2.50	10.07	1
12	2.00	1.61	1.00	0.00	2.97	2.00	1.00	10.58	1
25	2.00	2.18	1.00	0.00	1.02	2.00	2.50	10.70	1
19	2.00	1.91	1.00	0.00	2.95	2.00	1.00	10.86	2
3	2.00	1.94	1.00	0.00	1.12	2.00	3.00	11.07	2
11	2.00	3.17	1.00	0.00	1.01	2.00	2.00	11.17	2
21	3.00	2.21	1.00	0.00	1.00	2.00	2.00	11.21	2
9	3.00	1.94	1.00	0.00	2.97	2.00	1.00	11.91	2
8	2.00	2.46	1.00	0.00	1.17	2.00	3.50	12.12	2
20	3.00	1.88	1.00	0.00	2.98	2.00	1.50	12.36	3
23	2.00	2.28	1.00	0.00	3.06	2.00	2.50	12.84	3
2	1.00	1.85	1.00	0.00	3.23	2.00	4.00	13.08	3
14	2.00	1.45	1.00	0.00	3.19	2.00	3.50	13.15	3
24	2.00	2.51	1.00	0.00	3.11	2.00	3.00	13.62	3
4	2.00	3.13	1.00	0.00	1.31	2.00	4.50	13.94	3
1	2.00	2.22	1.00	2.00	1.22	2.00	4.00	14.44	3
10	2.00	3.08	3.11	0.00	3.13	2.00	3.50	16.82	4
16	2.00	2.60	3.09	0.00	3.23	2.00	4.00	16.93	4
7	2.00	4.42	3.30	0.00	3.51	2.00	5.00	20.22	4
17	3.00	4.62	3.17	2.00	5.40	4.00	4.50	26.69	4
6	3.00	7.28	3.27	4.00	5.67	4.00	5.00	32.23	4

Villages listed in order of increasing urbanicity score (total points).

### Urbanicity and Cardiometabolic Risk Factors

Of the 7,830 individuals aged 13 y and above surveyed during round 22 of the GPC, 490 individuals were excluded due to incomplete data (21 participants were no longer in the GPC dataset and 469 participants did not have any SES data). The final study population was therefore 7,340 residents in the 25 villages in Kyamulibwa, representing 95% of the total study population in round 22 of the GPC. The characteristics of this population stratified by sex and quartile of urbanicity are presented in Table 4. Residents in the most urban villages were younger (mean age, 33 y), predominantly Ugandan (83%), and more likely to be educated beyond primary school level (41%), compared to those resident in the least urban villages (mean age, 35 y; 77% Ugandan; 17% educated beyond primary school level). There were fewer unemployed men but more unemployed women in the most urban villages compared to the least urban villages (Table 4).

**Table 4:** Characteristics of study participants by sex, General Population Cohort, Uganda, 2011.

Population	Characteristic	Urbanicity Level											
		Quartile 1 (Least Urban)			Quartile 2			Quartile 3			Quartile 4 (Most Urban)		
		Number	Percent	Mean (SD)	Number	Percent	Mean (SD)	Number	Percent	Mean (SD)	Number	Percent	Mean (SD)
**Total**	**Number of participants**	1,878			1,901			1,926			1,635		
	**Age in years**			35.2 (18.9)			34.6 (18.6)			34.5 (18.7)			32.7 (16.4)
	**Ethnic origin**												
	Ugandan	1,427	77.2		1,457	77.4		1,561	81.9		1,335	82.7	
	Non-Ugandan	422	22.8		425	22.6		346	18.1		280	17.3	
	**Highest level of education achieved**												
	Primary school or less	1,561	83.1		1,491	78.4		1,424	73.9		964	59.0	
	Higher than primary school	317	16.9		410	21.6		502	26.1		671	41.0	
	**Employment status**												
	Employed	1,297	91.3		1,239	89.1		1,236	91.3		1,106	89.5	
	Unemployed	124	8.7		151	10.9		118	8.7		130	10.5	
	**SES**			−0.18 (1.6)			−0.10 (1.6)			−0.04 (1.7)			0.38 (1.5)
**Men**	**Number of participants**	820	43.6		824	43.3		859	44.6		712	43.5	
	**Age in years**			33.9 (19.4)			32.9 (18.8)			33.5 (19.4)			31.5 (15.8)
	**Ethnic origin**												
	Ugandan	609	75.6		633	77.8		672	79.2		576	81.9	
	Non-Ugandan	198	24.5		181	22.2		177	20.8		127	18.1	
	**Highest level of education achieved**												
	Primary school or less	679	82.8		650	78.9		636	74.0		430	60.4	
	Higher than primary school	141	17.2		174	21.1		223	26.0		282	39.6	
	**Employment status**												
	Employed	554	94.1		527	94.1		533	94.7		490	96.5	
	Unemployed	35	5.9		33	5.9		30	5.3		18	3.5	
	**SES**			−0.28 (1.7)			−0.15 (1.6)			−0.04 (1.8)			0.33 (1.5)
**Women**	**Number of participants**	1,058	56.3		1,077	56.6		1,067	55.4		923	56.5	
	**Age in years**			36.2 (18.5)			35.9 (18.3)			35.4 (18.1)			33.6 (16.7)
	**Ethnic origin**												
	Ugandan	818	78.5		824	77.2		889	84.0		759	83.2	
	Non-Ugandan	224	21.5		244	22.8		169	16.0		153	16.8	
	**Highest level of education achieved**												
	Primary school or less	882	83.4		841	78.1		788	73.8		534	57.8	
	Higher than primary school	176	16.6		236	21.9		279	26.1		389	42.2	
	**Employment status**												
	Employed	743	89.3		712	85.8		703	88.9		616	84.6	
	Unemployed	89	10.7		118	14.2		88	11.1		112	15.4	
	**SES**			−0.11 (1.5)			−0.07 (1.6)			−0.04 (1.6)			0.41 (1.6)

Table 5 presents the prevalence of lifestyle risk factors by sex and quartile of urbanicity. Prevalence of most lifestyle risk factors, including low fruit and vegetable intake, low physical activity, high BMI, and high WC, were higher among residents in the most urban villages compared with those in the least urban settlements (Table 5). In the most urban villages a larger proportion of women were physically inactive (72%), had high BMI (22%), and had high WC (33%) compared with the men in the same villages (58% physically inactive, 8% with high BMI, and 2% with high WC). Unadjusted and partially adjusted (adjusted for clustering and all confounders except SES) models of associations between individual lifestyle risk factors and quartile of urbanicity stratified by sex are shown in Tables S1 and S2, respectively.

**Table 5:** Prevalence of lifestyle risk factors for cardiometabolic diseases by sex, General Population Cohort, Uganda, 2011.

Lifestyle Risk Factor	Urbanicity Level							
	Quartile 1 (Least Urban)		Quartile 2		Quartile 3		Quartile 4 (Most Urban)	
	Number	Percent	Number	Percent	Number	Percent	Number	Percent
**Total**								
Current smoking	170	9.1	154	8.1	162	8.4	117	7.2
Heavy drinking^a^	9	0.5	16	0.8	15	0.8	18	1.1
Low fruit and vegetable consumption^b^	1,292	69.1	1,527	80.6	1,425	74.2	1,339	82.3
Low physical activity^c^	1,050	55.9	1,133	59.6	1,132	58.8	1,074	65.7
High BMI^d^	187	10.3	215	11.7	217	11.6	249	15.8
High WC^e^	295	16.2	346	18.9	309	16.5	304	19.2
High BP^f^	330	17.6	313	16.5	313	16.3	247	15.1
**Men**								
Current smoking	151	18.4	129	15.7	139	16.2	99	13.9
Heavy drinking^a^	5	0.6	8	1.0	11	0.4	12	1.7
Low fruit and vegetable consumption^b^	561	68.7	653	79.4	610	71.3	580	81.9
Low physical activity^c^	409	49.9	422	51.2	444	51.7	411	57.8
High BMI^d^	30	3.7	40	4.9	39	4.6	54	7.6
High WC^e^	7	0.9	8	1.0	15	1.8	16	2.3
High BP^f^	138	16.9	134	16.3	137	16.0	117	16.5
**Women**								
Current smoking	19	1.8	25	2.3	23	2.2	18	1.9
Heavy drinking^a^	4	0.4	8	0.7	4	1.2	6	0.7
Low fruit and vegetable consumption^b^	731	69.3	874	81.4	815	76.4	759	82.5
Low physical activity^c^	641	60.6	711	66.0	688	64.5	663	71.8
High BMI^d^	157	15.6	175	17.2	178	17.5	195	22.3
High WC^e^	288	28.7	338	33.4	294	29.0	288	32.9
High BP^f^	192	18.2	179	16.7	176	16.5	130	14.1

a Heavy drinking defined as any woman who reports drinking more than one drink per day or any man who reports drinking more than two drinks per day.

b Low fruit and vegetable consumption defined as eating less than five portions of fruit or vegetables per day.

c Low physical activity defined as doing less than 5 d a week of any combination of walking or moderate- or vigorous-intensity activities and less than 600 min of physical activity per week.

d High BMI defined as BMI≥25 kg/m^2^.

e High WC defined as WC≥94 cm for men and WC≥80 cm for women.

f High BP defined as systolic BP≥140 mm Hg or diastolic BP≥90 mm Hg or reported treatment for high BP.

Table 6 shows results from fully adjusted models stratified by sex. Overall, we observed that increasing levels of urbanicity were associated with lifestyle risk factors for cardiometabolic diseases even after adjusting for clustering and potential confounders including SES. Specifically, men in the most urban villages had a higher risk of heavy drinking (risk ratio [RR]: 3.95; 95% CI: 1.40, 11.13), low fruit and vegetable consumption (RR: 1.20; 95% CI: 1.12, 1.27), low physical activity (RR: 1.15; 95% CI: 1.04, 1.27), high BMI (RR: 1.98; 95% CI: 1.28, 3.06), and high WC (RR: 2.86; 95% CI: 1.19, 6.82) than those in the least urban villages, even after adjusting for SES, age, and clustering at household level. Apart from the association of urbanicity with heavy drinking and high WC, similar results were observed among women (Table 6). No association was found between urbanicity and current smoking or high BP among men or women.

**Table 6:** Associations between increasing urbanicity and lifestyle risk factors adjusted for age, socioeconomic status, and clustering at household level, General Population Cohort, Uganda, 2011.

Lifestyle Risk Factor	Urbanicity Level				*p*-Value (Test for Non-Linearity)▴	*p*-Value (Interaction)
	Quartile 1 (Least Urban)	Quartile 2	Quartile 3	Quartile 4 (Most Urban)		
	RR	RR (95% CI)	RR (95% CI)	RR (95% CI)		
**Total** †						
Current smoking	1	0.96 (0.78, 1.17)	0.96 (0.79, 1.16)	1.10 (0.88, 1.36)	0.71	0.72
Heavy drinking^a^	1	1.91 (0.82, 4.46)	1.67 (0.71, 3.89)	3.33* (1.43, 7.82)	0.55	0.59
Low fruit and vegetable consumption^b^	1	1.17** (1.12, 1.22)	1.07* (1.02, 1.13)	1.19** (1.14, 1.24)	0.001*	0.83
Low physical activity^c^	1	1.06* (1.00, 1.13)	1.05 (0.99, 1.11)	1.17** (1.10, 1.23)	0.68	0.48
High BMI^d^	1	1.14 (0.95, 1.37)	1.13 (0.93, 1.37)	1.48** (1.24, 1.77)	0.82	0.45
High WC^e^	1	1.17* (1.02, 1.33)	1.05 (0.91, 1.21)	1.21* (1.05, 1.38)	0.18	0.10
High BP^f^	1	0.95 (0.83, 1.08)	0.93 (0.81, 1.06)	0.97 (0.84, 1.12)	0.64	0.28
**Men**						
Current smoking	1	0.91 (0.74, 1.12)	0.92 (0.76, 1.13)	1.05 (0.84, 1.32)	0.80	—
Heavy drinking^a^	1	1.71 (0.56, 5.22)	2.26 (0.79, 6.45)	3.95* (1.40, 11.13)	0.43	—
Low fruit and vegetable consumption^b^	1	1.16** (1.08, 1.23)	1.04 (0.97, 1.11)	1.20** (1.12, 1.27)	0.02*	—
Low physical activity^c^	1	1.02 (0.92, 1.13)	1.03 (0.94, 1.14)	1.15* (1.04, 1.27)	0.55	—
High BMI^d^	1	1.32 (0.83, 2.08)	1.16 (0.73, 1.86)	1.98* (1.28, 3.06)	0.95	—
High WC^e^	1	1.15 (0.43, 3.05)	1.89 (0.76, 4.68)	2.86* (1.19, 6.82)	0.13	—
High BP^f^	1	0.97 (0.79, 1.18)	0.92 (0.75, 1.12)	1.09 (0.88, 1.34)	0.90	—
**Women**						—
Current smoking	1	1.29 (0.72, 2.31)	1.24 (0.67, 2.29)	1.43 (0.75, 2.75)	0.80	—
Heavy drinking^a^	1	1.95 (0.59, 6.42)	1.02 (0.26, 4.04)	2.26 (0.62, 8.17)	0.43	—
Low fruit and vegetable consumption^b^	1	1.17** (1.11, 1.24)	1.10* (1.04, 1.17)	1.19** (1.12, 1.25)	0.02*	—
Low physical activity^c^	1	1.09* (1.02, 1.16)	1.06 (0.99, 1.14)	1.17** (1.09, 1.25)	0.55	—
High BMI^d^	1	1.10 (0.90, 1.34)	1.12 (0.92, 1.37)	1.39* (1.15, 1.68)	0.94	—
High WC^e^	1	1.17* (1.02, 1.33)	1.03 (0.89, 1.18)	1.18 (1.02, 1.35)	0.13	—
High BP^f^	1	0.93 (0.78, 1.11)	0.94 (0.79, 1.12)	0.88 (0.72, 1.07)	0.90	—

▴ Test for non-linearity based on likelihood ratio tests comparing models with urbanicity as a categorical and as a continuous variable, with the former model nested in the latter.

† All estimates for the total population were also adjusted for sex.

a Heavy drinking defined as any woman who reports drinking more than one drink per day or any man who reports drinking more than two drinks per day.

b Low fruit and vegetable consumption defined as eating less than five portions of fruit or vegetables per day.

c Low physical activity defined as doing less than 5 d a week of any combination of walking or moderate- or vigorous-intensity activities and less than 600 min of physical activity per week.

d High BMI defined as BMI≥25 kg/m^2^.

e High WC defined as WC≥94 cm for men and WC≥80 cm for women.

f High BP defined as systolic BP≥140 mm Hg or diastolic BP≥90 mm Hg or reported treatment for high BP. Also adjusted for BMI.

^*^ *p*<0.05.

**p<0.001.

Results from our sensitivity analyses indicate that associations observed were broadly consistent when lifestyle risk factors and urbanicity were considered as continuous and categorical variables (Tables S3–S5). There was no clear or consistent evidence of non-linearity of associations across the models used in our primary and sensitivity analyses (Tables 6, S3, and S5). In addition, there was no statistical evidence of any interaction between sex and urbanicity on lifestyle risk factors in our study population following post hoc analysis.

With regards to missing data, there were no differences in the proportion of participants with missing lifestyle risk factor data by quartile of urbanicity (Table S6). Although the majority of participants with missing SES data resided in the most urban villages, there were no differences in demographic characteristics or the prevalence of lifestyle risk factors between those who were included and excluded from our analyses because of missing SES data (Table S7). With regards to the urbanicity scale, less than 6% of adults in round 22 of the GPC did not have information on their primary occupation. Overall, of those missing primary occupation data, a smaller proportion were current smokers, were heavy drinkers, had high BMI, had high WC, and had high BP compared to those with primary occupation data (Table S8). However, sensitivity analyses show that our results do not materially change when all or none of those with missing primary occupation data are presumed to be involved in primary agriculture (Tables S9 and S10).

## Discussion

Across 25 rural villages in Uganda, we found a marked variation in levels of urbanicity, with strong evidence that lifestyle risk factors were associated with increasing urbanicity levels among men and women. Our findings not only challenge the prevailing use of dichotomous urban–rural classification systems in epidemiological studies, but also indicate that even small-scale increases in urbanicity levels across rural environments are associated with a higher prevalence of unhealthy behaviours among rural residents. Considering that lifestyle risk factors are potentially modifiable, these findings may have important implications for approaches to prevention of cardiometabolic diseases as rural populations and environments increasingly adopt urban characteristics.

Our findings are consistent with previous studies that have examined the relationship between behavioural risk factors and urban living. Several studies to date have used scale-based definitions to evaluate urbanicity as an exposure for cardiometabolic risk factors [11]–[16]. These studies have found that a multi-component scale-based definition of urbanicity better captured relations between urbanicity and health than dichotomous definitions [11]–[16],[18],[40]. In addition, they all reported strong evidence of associations between increasing urbanicity levels and physical inactivity, high BMI, diabetes mellitus, or hypertension [11]–[16],[18],[40]. Studies using dichotomous comparisons of urbanicity have also reported an association between length of time exposed to urban environments and common modifiable risk factors for cardiometabolic diseases across LMICs [41],[42]. For example, a recent cross-sectional study in India examining the association between urban life-years and cardiometabolic risk in 4,221 individuals found an association between increasing urban life-years and an initial rise in adiposity followed by a more gradual and sustained increase in BP and insulin [43],[44]. Another cross-sectional study in Cameroon reported that migrating to urban areas was associated with higher fasting blood sugar, higher BMI, and higher BP compared to rural residents with less than 2 y of exposure to urban environments [45]. Although we could not assess the effects of time spent in more urban environments on cardiometabolic risk, our results are consistent with previous research and show that even in rural settings, living in areas with more urban characteristics is associated with an increase in unhealthy behaviours, including low fruit and vegetable consumption, reduced physical activity, and having higher BMI, even after adjusting for SES.

The urbanicity score in our study captures urban features such as reduction in agricultural occupations, access to more services, and longer time spent in education. Interestingly, although residents in the most urban villages had diets low in fruit and vegetables, reduced physical activity, and increased BMIs, they did not have high BP. This finding requires further exploration. Previous studies have suggested that as areas adopt urban characteristics, energy consumption and expenditure patterns of populations change through a rise in energy-rich diets and sedentary lifestyles [43],[46]. This in turn may result in a rapid increase in adiposity among exposed populations, while the effects of urbanicity on BP are observed only over longer periods of time, as initial increases in adiposity stabilize [43],[46]. A study in India, for example, estimated a 1-mm Hg increase in BP per decade of exposure to urban environments [43]. And a study of 200 participants in Benin found that age- and sex-adjusted odds ratios for hypertension were 1.3 and 2.8 among individuals who had spent 21–33 y and ≥34 y, respectively, in a city compared to those who had spent ≤20 y in a city [6]. Currently, however, there are very limited data exploring the magnitude of associations between BMI and BP over time within African populations.

To our knowledge, this is the first study in SSA to examine lifestyle risk factors for cardiometabolic diseases in relation to varying degrees of urbanicity in rural areas. Unlike the widely used dichotomous urban–rural classification system, we used a scale-based measure of urbanicity that was sensitive enough to detect variations in levels of urbanicity across rural settlements. In addition to the large study sample size, individual-level demographic, lifestyle, and biophysical data were aligned to village-level urbanicity information, reducing the possibility of ecological fallacies.

Our study is limited by its cross-sectional study design, which restricts the conclusions that can be drawn about the temporal relationship between changing environments and the increase in cardiometabolic risk in rural populations. Another potential limitation was recall bias, as some risk factor data were based on self-reported measures; however, the influence of recall bias was minimized by blinding participants to the study exposure and using data collection tools based on validated questionnaires [21]. To reduce the possibility of a chance association due to multiple testing, only widely accepted lifestyle risk factors, with known biological mechanisms for increasing cardiometabolic risk, were selected a priori for analyses. Results from our primary analyses were consistent with our sensitivity analyses; moreover, all our associations were directionally consistent with previous studies in other LMICs including China, the Philippines, India, and Sri Lanka [11]–[16],[18],[40]. Although we found no clear evidence of non-linearity, the detailed shape of associations between urbanicity and lifestyle risk factors will require further investigation in studies with appropriate statistical resolution. With regards to missing data, individuals excluded because of missing data were representative of the study population. Sensitivity analyses also showed that individuals’ missing primary occupation data related to the urbanicity scale did not change the results observed.

Although there were two minor adjustments to our urbanicity score from that of Novak et al. [14], related to indicators of SES [22]–[26], results from our analyses show that inclusion of SES in our models does not materially change the results observed. The interrelation between SES and urbanicity is complex and beyond the scope of this study; however, directions of all associations observed are consistent with previous literature [11]–[16],[18],[40],[47]. Finally the generalisability of our results to other rural communities needs to be considered. Although there is a lack of comparable data across other rural areas in Africa, broad consistency of our findings with previous research argues against limitations of generalisability [41],[48].

## Conclusion

SSA remains the least urbanized region in the world, with the majority of the population residing in rural areas and economies still heavily dependent on subsistence agriculture [49]. However, through globalization and technological advances, the boundaries between urban and rural settlements are becoming more obscure [49]. With the growing complexity of human settlements, dichotomous classification systems based on a single parameter could become increasingly inadequate for capturing information needed to develop targeted strategies for disease management and control in the context of urbanization in rural areas [12],[18]. Our results indicate associations between increasing urbanicity levels and unhealthy lifestyles in rural communities. This is an important finding, considering that over 533 million people live in rural areas across SSA [19] and that any increase in cardiometabolic risk associated with the development process in these areas is likely to have an impact on population health and healthcare services. In these contexts, it will be important to explore how cardiometabolic risks accrue in relation to amount of time spent in more urban environments and also to assess differences in these associations using more objective measures of lifestyle risk factors [50]. Future studies should also examine factors influencing differential access to healthcare services among population sub-groups resident in a single area. A better understanding of these associations is crucial because modification of lifestyle risk factors through changes in the physical environment, including local infrastructure, may provide a potential avenue for primary prevention of cardiometabolic diseases in rural populations.

## Supporting Information

Table S1 Unadjusted estimates of associations between increasing urbanicity and lifestyle risk factors, General Population Cohort, Uganda, 2011.
(DOCX)

Table S2 Associations between increasing urbanicity and lifestyle risk factors adjusted for age, sex, and clustering at household level, General Population Cohort, Uganda, 2011.
(DOCX)

Table S3 Differences in the means of each lifestyle risk factor by urbanicity quartile adjusted for age, sex, socioeconomic status, and clustering at household level, General Population Cohort, Uganda, 2011.
(DOCX)

Table S4 Associations between a 1–standard deviation change in urbanicity and lifestyle risk factors adjusted for age, sex, clustering at household level, and socioeconomic status, Uganda, 2011.
(DOCX)

Table S5 Unadjusted and adjusted associations between a 1–standard deviation change in urbanicity and lifestyle risk factors by sex, Uganda, 2011.
(DOCX)

Table S6 Numbers and proportions of participants with missing data by urbanicity quartile, General Population Cohort, Uganda, 2011.
(DOCX)

Table S7 Characteristics and prevalence of lifestyle risk factors among those with and without socioeconomic status data overall and by urbanicity quartile, General Population Cohort, Uganda, 2011.
(DOCX)

Table S8 Characteristics and prevalence of lifestyle risk factors among participants aged ≥18 y with and without primary occupation data overall and by urbanicity quartile, General Population Cohort, Uganda, 2011.
(DOCX)

Table S9 Associations between increasing urbanicity and lifestyle risk factors adjusted for age, socioeconomic status, and clustering at household level, assuming those with missing primary occupation data are all involved or not involved in agriculture, General Population Cohort, Uganda, 2011.
(DOCX)

Table S10 Associations between a 1–standard deviation change in urbanicity and lifestyle risk factors adjusted for age, socioeconomic status, and clustering at household level, assuming those with missing primary occupation data are all involved or not involved in agriculture, General Population Cohort, Uganda, 2011.
(DOCX)

## Abbreviations

BMI: body mass index
BP: blood pressure
CI: confidence interval
GPC: General Population Cohort
LMICs: low- and middle-income countries
MRC/UVRI: Medical Research Council/Uganda Virus Research Institute
PCA: principal component analysis
RR: risk ratio
SD: standard deviation
SES: socioeconomic status
SSA: sub-Saharan Africa
WC: waist circumference
