# Supplementary material for: Urbanicity and Lifestyle Risk Factors for Cardiometabolic Diseases in Rural Uganda: A Cross-Sectional Study
Source: PLoS Med. 2014 Jul 29;11(7):e1001683. doi: 10.1371/journal.pmed.1001683 (PMC4114555; doi:10.1371/journal.pmed.1001683)
Supplement: Table S1 — Unadjusted estimates of associations between increasing urbanicity and lifestyle risk factors, General Population Cohort, Uganda, 2011. (DOCX) [file pmed.1001683.s001.docx]

**Table S1. Unadjusted estimates of associations between increasing urbanicity and lifestyle risk factors, General Population Cohort, Uganda, 2011**

|  | Urbanicity level | | | | | | |
| --- | --- | --- | --- | --- | --- | --- | --- |
| Lifestyle Risk Factor | Quartile 1  (least urban) |  | Quartile 2 |  | Quartile 3 |  | Quartile 4  (most urban) |
|  | RR |  | RR (95%CI) |  | RR (95%CI) |  | RR (95%CI) |
| Total |  |  |  |  |  |  |  |
| Current smokers | 1 |  | 0.89 (0.72, 1.11) |  | 0.93 (0.75, 1.15) |  | 0.79 (0.62, 1.00) |
| Heavy drinkers ^a^ | 1 |  | 1.76 (0.78, 3.97) |  | 1.63 (0.71, 3.71) |  | 2.30* (1.03, 5.11) |
| Low fruit and vegetable consumption ^b^ | 1 |  | 1.17** (1.08, 1.26) |  | 1.07 (0.99, 1.16) |  | 1.19** (1.10, 1.28) |
| Low physical activity ^c^ | 1 |  | 1.06 (0.98, 1.16) |  | 1.05 (0.97, 1.14) |  | 1.17** (1.08, 1.28) |
| High BMI ^d^ | 1 |  | 1.14 (0.94, 1.38) |  | 1.13 (0.93, 1.37) |  | 1.53** (1.27, 1.85) |
| Abdominal obesity ^e^ | 1 |  | 1.16 (0.99, 1.36) |  | 1.02 (0.87, 1.19) |  | 1.18* (1.01, 1.39) |
| High BP ^f^ | 1 |  | 0.94 (0.80, 1.09) |  | 0.93 (0.79, 1.08) |  | 0.86 (0.72, 1.01) |
| Men |  |  |  |  |  |  |  |
| Current smokers | 1 |  | 0.85 (0.67, 1.07) |  | 0.88 (0.70, 1.11) |  | 0.75* (0.59, 0.97) |
| Heavy drinkers ^a^ | 1 |  | 1.59 (0.52, 4.87) |  | 2.10 (0.73, 6.04) |  | 2.76 (0.97, 7.84) |
| Low fruit and vegetable consumption ^b^ | 1 |  | 1.16* (1.03, 1.30) |  | 1.04 (0.93, 1.16) |  | 1.19* (1.06, 1.34) |
| Low physical activity ^c^ | 1 |  | 1.03 (0.90, 1.18) |  | 1.04 (0.90, 1.18) |  | 1.16* (1.01, 1.33) |
| High BMI ^d^ | 1 |  | 1.32 (0.82, 2.13) |  | 1.24 (0.77, 1.99) |  | 2.07* (1.33, 3.24) |
| WC ^e^ | 1 |  | 1.13 (0.41, 3.13) |  | 2.04 (0.83, 5.01) |  | 2.63* (1.08, 6.40) |
| High BP ^f^ | 1 |  | 0.96 (0.76, 1.22) |  | 0.95 (0.75, 1.20) |  | 0.97 (0.76, 1.25) |
| Women |  |  |  |  |  |  |  |
| Current smokers | 1 |  | 1.29 (0.71, 2.35) |  | 1.20 (0.65, 2.20) |  | 1.08 (0.57, 2.07) |
| Heavy drinkers ^a^ | 1 |  | 1.96 (0.59, 6.52) |  | 0.99 (0.25, 3.96) |  | 1.72 (0.48, 6.09) |
| Low fruit and vegetable consumption ^b^ | 1 |  | 1.17* (1.06, 1.29) |  | 1.10 (0.99, 1.22) |  | 1.19* (1.07, 1.32) |
| Low physical activity ^c^ | 1 |  | 1.09 (0.98, 1.21) |  | 1.06 (0.95, 1.18) |  | 1.18* (1.06, 1.32) |
| High BMI ^d^ | 1 |  | 1.10 (0.89, 1.37) |  | 1.12 (0.90, 1.38) |  | 1.43* (1.16, 1.76) |
| WC ^e^ | 1 |  | 1.16 (0.99, 1.36) |  | 1.01 (0.86, 1.19) |  | 1.15 (0.97, 1.35) |
| High BP ^f^ | 1 |  | 0.92 (0.75, 1.12) |  | 0.91 (0.74, 1.11) |  | 0.77 (0.62, 0.97) |

Abbreviations: BMI, body mass index; BP, blood pressure; CI, confidence interval; RR, risk ratio.

^a^ Heavy drinkers defined as any woman who reports drinking more than one drink per day or any man who reports drinking more than two drinks per day.

^b^ Low fruit and vegetable consumption defined as eating less than five portions of fruit or vegetables per day

^c^ Low physical activity defined as doing less than 5 days a week of any combination of walking, moderate or vigorous intensity activities and less than 600 minutes of physical activity per week

^d^ High BMI defined as BMI ≥ 25kg/m^2^

^e^ Abdominal obesity defined as waist circumference ≥94 cm for men and ≥80 cm for women

^f^ High BP defined as blood pressure ≥140/90 mmHg or reported treatment for high blood pressure

* *P* <0.05

** *P* <0.001
