# Supplementary material for: Urbanicity and Lifestyle Risk Factors for Cardiometabolic Diseases in Rural Uganda: A Cross-Sectional Study
Source: PLoS Med. 2014 Jul 29;11(7):e1001683. doi: 10.1371/journal.pmed.1001683 (PMC4114555; doi:10.1371/journal.pmed.1001683)
Supplement: Table S2 — Associations between increasing urbanicity and lifestyle risk factors adjusted for age, sex, and clustering at household level, General Population Cohort, Uganda, 2011. (DOCX) [file pmed.1001683.s002.docx]

**Table S2. Associations between increasing urbanicity and lifestyle risk factors adjusted for age, sex, and clustering at household level, General Population Cohort, Uganda, 2011**

| Lifestyle Risk Factor |  |  |  |  | Urbanicity level |  |  |
| --- | --- | --- | --- | --- | --- | --- | --- |
|  | Quartile 1 (least urban) |  | Quartile 2 |  | Quartile 3 |  | Quartile 4 (most urban) |
|  | RR |  | RR (95%CI) |  | RR (95%CI) |  | RR (95%CI) |
| Total^†^ |  |  |  |  |  |  |  |
| Current smokers | 1 |  | 0.94 (0.77, 1.16) |  | 0.93 (0.76, 1.14) |  | 0.95 (0.76, 1.18) |
| Heavy drinkers ^a^ | 1 |  | 1.88 (0.81, 4.37) |  | 1.63 (0.69, 3.85) |  | 2.73* (1.18, 6.34) |
| Low fruit and vegetable consumption ^b^ | 1 |  | 1.17** (1.11, 1.22) |  | 1.07* (1.02, 1.13) |  | 1.19** (1.14, 1.25) |
| Low physical activity ^c^ | 1 |  | 1.06* (1.00, 1.13) |  | 1.05 (0.99, 1.11) |  | 1.17** (1.10, 1.24) |
| High BMI ^d^ | 1 |  | 1.15 (0.95, 1.38) |  | 1.15 (0.95, 1.40) |  | 1.61** (1.34, 1.92) |
| Abdominal obesity ^e^ | 1 |  | 1.17* (1.02, 1.34) |  | 1.06 (0.92, 1.22) |  | 1.27* (1.11, 1.45) |
| High BP ^f ◊^ | 1 |  | 0.95 (0.84, 1.08) |  | 0.93 (0.82, 1.06) |  | 0.99 (0.86, 1.14) |
| Men |  |  |  |  |  |  |  |
| Current smokers | 1 |  | 0.89 (0.72, 1.10) |  | 0.89 (0.73, 1.10) |  | 0.91 (0.72, 1.14) |
| Heavy drinkers ^a^ | 1 |  | 1.65 (0.54, 5.05) |  | 2.12 (0.74, 6.11) |  | 3.18* (1.12, 8.99) |
| Low fruit and vegetable consumption ^b^ | 1 |  | 1.16** (1.09, 1.23) |  | 1.04 (0.97, 1.11) |  | 1.20** (1.12, 1.28) |
| Low physical activity ^c^ | 1 |  | 1.03 (0.93, 1.14) |  | 1.04 (0.94, 1.14) |  | 1.16* (1.05, 1.28) |
| High BMI ^d^ | 1 |  | 1.36 (0.85, 2.15) |  | 1.24 (0.78, 1.99) |  | 2.24** (1.45, 3.47) |
| WC ^e^ | 1 |  | 1.20 (0.44, 3.26) |  | 2.08 (0.86, 5.00) |  | 3.32* (1.40, 7.85) |
| High BP ^f ◊^ | 1 |  | 0.97 (0.79, 1.18) |  | 0.92 (0.75, 1.13) |  | 1.11 (0.89, 1.37) |
| Women |  |  |  |  |  |  |  |
| Current smokers | 1 |  | 1.32 (0.73, 2.39) |  | 1.27 (0.68, 2.35) |  | 1.27 (0.67, 2.41) |
| Heavy drinkers ^a^ | 1 |  | 2.00 (0.60, 6.64) |  | 1.04 (0.26, 4.16) |  | 1.96 (0.56, 6.91) |
| Low fruit and vegetable consumption ^b^ | 1 |  | 1.17** (1.11, 1.23) |  | 1.10* (1.04, 1.17) |  | 1.19** (1.13, 1.25) |
| Low physical activity ^c^ | 1 |  | 1.09* (1.02, 1.16) |  | 1.06 (0.99, 1.14) |  | 1.18** (1.10, 1.26) |
| High BMI ^d^ | 1 |  | 1.11 (0.91, 1.35) |  | 1.13 (0.93, 1.38) |  | 1.49** (1.23, 1.80) |
| WC ^e^ | 1 |  | 1.17* (1.02, 1.34) |  | 1.03 (0.89, 1.19) |  | 1.22* (1.07, 1.40) |
| High BP ^f ◊^ | 1 |  | 0.93 (0.78, 1.11) |  | 0.94 (0.79, 1.12) |  | 0.88 (0.73, 1.07) |

Abbreviations: BMI, body mass index; BP, blood pressure; CI, confidence interval; RR, risk ratio.

**^†^** All estimates for the total population were also adjusted for sex.

^a^ Heavy drinkers defined as any woman who reports drinking more than one drink per day or any man who reports drinking more than two drinks per day.

^b^ Low fruit and vegetable consumption defined as eating less than five portions of fruit or vegetables per day

^c^ Low physical activity defined as doing less than 5 days a week of any combination of walking, moderate or vigorous intensity activities and less than 600 minutes of physical activity per week

^d^ High BMI defined as BMI ≥ 25kg/m^2^

^e^ Abdominal obesity defined as waist circumference ≥94 cm for men and ≥80 cm for women

^f^ High BP defined as blood pressure ≥140/90 mmHg or reported treatment for high blood pressure

**^◊^** Also adjusted for BMI

* *P* <0.05

** *P* <0.001
