# Supplementary material for: Urbanicity and Lifestyle Risk Factors for Cardiometabolic Diseases in Rural Uganda: A Cross-Sectional Study
Source: PLoS Med. 2014 Jul 29;11(7):e1001683. doi: 10.1371/journal.pmed.1001683 (PMC4114555; doi:10.1371/journal.pmed.1001683)
Supplement: Table S3 — Differences in the means of each lifestyle risk factor by urbanicity quartile adjusted for age, sex, socioeconomic status, and clustering at household level, General Population Cohort, Uganda, 2011. (DOCX) [file pmed.1001683.s003.docx]

**Table S3. Differences in the means of each lifestyle risk factor by urbanicity quartile adjusted for age, sex, socioeconomic status, and clustering at household level, General Population Cohort, Uganda, 2011**

| Lifestyle Risk Factor | Urbanicity level | | | |  |
| --- | --- | --- | --- | --- | --- |
|  | Quartile 1 (least urban) | Quartile 2 | Quartile 3 | Quartile 4 (most urban) | P-value (test for non-linearity) ^▲^ |
|  | Mean  (Reference) | Difference in mean (95%CI) | Difference in mean (95%CI) | Difference in mean (95%CI) |  |
| Total^†^ |  |  |  |  |  |
| Alcohol intake (number of drinks per day) ^▲^ | 0.29 | 0.98 (-0.01, 0.21) | 0.10 (-0.03, 0.23) | 0.22** (0.10, 0.34) | 0.61 |
| Fruit and vegetable consumption (number per day) | 4.11 | -1.02** (-1.23, -0.80) | -0.36* (-0.59, -0.12) | -1.11** (-1.34, -0.88) | <0.001 |
| Physical activity (number of minutes per week) | 833.93 | -71.36* (-134.39, -6.33) | -49.15 (-113.51, 15.21) | -136.74** (-204.59, -68.89) | 0.29 |
| BMI | 20.92 | 0.26 (-0.00, 0.51) | 0.10 (-0.15, 0.36) | 0.66** (0.38, 0.94) | 0.20 |
| WC | 74.57 | 0.60* (0.04, 1.17) | 0.04 (-0.51, 0.59) | 1.33** (0.71, 1.96) | 0.04 |
| SBP (mmHg) ^◊^ | 123.16 | -0.69 (-1.74, 0.35) | -0.49 (-1.57, 0.58) | -0.82 (-1.89, 0.25) | 0.55 |
| DBP (mmHg) ^◊^ | 74.72 | -0.50 (-1.14, 0.14) | -1.24** (-1.92, -0.55) | 0.12 (-0.55, 0.79) | <0.001 |
| Men |  |  |  |  |  |
| Alcohol intake (number of drinks per day) | 0.39 | 0.15 (-0.01, 0.31) | 0.17 (-0.01, 0.35) | 0.27* (0.11, 0.43) | - |
| Fruit and vegetable consumption (number per day) | 4.11 | -0.92** (-1.22, -0.62) | -0.11 (-0.45, 0.22) | -1.09** (-1.42, -0.77) | - |
| Physical activity (number of minutes per week) | 989.78 | -35.70 (-144.72, 73.31) | -0.06 (-107.92, 107.79) | -78.17 (-194.83, 38.49) | - |
| BMI | 19.83 | 0.37* (0.09, 0.66) | 0.17 (-0.12, 0.47) | 0.51* (0.18, 0.84) | - |
| WC | 72.84 | 0.26 (-0.36, 0.89) | 0.02 (-0.59, 0.63) | 1.16* (0.45, 1.88) | - |
| SBP (mmHg) ^◊^ | 124.16 | -1.15 (-2.60, 0.30) | -0.87 (-2.36, 0.61) | -0.47 (-1.95, 0.99) | - |
| DBP (mmHg) ^◊^ | 73.99 | -0.71 (-1.65, 0.22) | -1.52* (-2.49, -0.55) | 0.33 (-0.64, 1.32) | - |
| Women |  |  |  |  |  |
| Alcohol intake (number of drinks per day) | 0.19 | 0.01 (-0.12, 0.15) | 0.003 (-0.14, 0.14) | 0.15* (0.01, 0.30) | - |
| Fruit and vegetable consumption (number per day) | 4.14 | -1.11** (-1.38, -0.84) | -0.58** (-0.88, -0.29) | -1.15** (-1.45, -0.86) | - |
| Physical activity (number of minutes per week) | 712.62 | -106.53* (-181.56, -35.51) | -90.00* (-164.12, 15.89) | -181.78** (-258.34, -105.21) | - |
| BMI | 21.79 | 0.17 (-0.19, 0.54) | 0.11 (-0.25, 0.48) | 0.72** (0.32, 1.13) | - |
| WC | 75.98 | 0.91* (0.08, 1.73) | 0.12 (-0.68, 0.93) | 1.35* (0.44, 2.25) | - |
| SBP (mmHg) ^◊^ | 122.41 | -0.57 (-1.98, 0.84) | -0.27 (-1.73, 1.19) | -1.15 (-2.60, 0.29) | - |
| DBP (mmHg) ^◊^ | 75.31 | -0.37 (-1.21, 0.47) | -1.02* (-1.92, -0.13) | -0.09 (-0.96, 0.78) | - |

Abbreviations: BMI, body mass index; WC, waist circumference; SBP, systolic blood pressure; DBP, diastolic blood pressure; CI, confidence interval.

^†^ All estimates for the total population were also adjusted for sex.

**^◊^** Also adjusted for BMI

^▲^ Test for non-linearity based on likelihood ratio tests comparing models with urbanicity as a categorical and as a continuous variable, with the former model nested in the latter.

* *P* <0.05

** *P* <0.00
