# Supplementary material for: Urbanicity and Lifestyle Risk Factors for Cardiometabolic Diseases in Rural Uganda: A Cross-Sectional Study
Source: PLoS Med. 2014 Jul 29;11(7):e1001683. doi: 10.1371/journal.pmed.1001683 (PMC4114555; doi:10.1371/journal.pmed.1001683)
Supplement: Table S4 — Associations between a 1–standard deviation change in urbanicity and lifestyle risk factors adjusted for age, sex, clustering at household level, and socioeconomic status, Uganda, 2011. (DOCX) [file pmed.1001683.s004.docx]

**Associations between a 1–standard deviation change in urbanicity and lifestyle risk factors adjusted for age, sex, clustering at household level, and socioeconomic status, Uganda, 2011.**

| Lifestyle Risk Factor |  | Adjusted for age, sex, SES, and household clustering |
| --- | --- | --- |
|  |  | RR (95%CI) |
| Total^†^ |  |  |
| Current smokers |  | 1.06 (0.97, 1.14) |
| Heavy drinkers ^a^ |  | 1.61** (1.29, 2.01) |
| Low fruit and vegetable consumption ^b^ |  | 1.05** (1.04, 1.06) |
| Low physical activity ^c^ |  | 1.05* (1.03, 1.07) |
| High BMI ^d^ |  | 1.18** (1.11, 1.25) |
| WC ^e^ |  | 1.06* (1.01, 1.11) |
| High BP ^f◊^ |  | 1.01 (0.96, 1.07) |
| Men |  |  |
| Current smokers |  | 1.06 (0.97, 1.15) |
| Heavy drinkers ^a^ |  | 1.68** (1.29, 2.17) |
| Low fruit and vegetable consumption ^b^ |  | 1.05** (1.04, 1.07) |
| Low physical activity ^c^ |  | 1.04* (1.00, 1.07) |
| High BMI ^d^ |  | 1.33** (1.18, 1.51) |
| WC ^e^ |  | 1.45* (1.15, 1.83) |
| High BP ^f◊^ |  | 1.04 (0.97, 1.13) |
| Women |  |  |
| Current smokers |  | 1.04 (0.83, 1.31) |
| Heavy drinkers ^a^ |  | 1.47* (1.01, 2.12) |
| Low fruit and vegetable consumption ^b^ |  | 1.04** (1.03, 1.06) |
| Low physical activity ^c^ |  | 1.05** (1.03, 1.07) |
| High BMI ^d^ |  | 1.15** (1.07, 1.22) |
| WC ^e^ |  | 1.05 (1.00, 1.09) |
| High BP ^f◊^ |  | 0.99 (0.92, 1.06) |

Abbreviations: BMI, body mass index; BP, blood pressure; CI, confidence interval; RR, risk ratio.

**^†^** All estimates for the total population were also adjusted for sex.

^a^ Heavy drinkers defined as any woman who reports drinking more than one drink per day or any man who reports drinking more than two drinks per day.

^b^ Low fruit and vegetable consumption defined as eating less than five portions of fruit or vegetables per day

^c^ Low physical activity defined as doing less than 5 days a week of any combination of walking, moderate or vigorous intensity activities and less than 600 minutes of physical activity per week

^d^ High BMI defined as BMI ≥ 25kg/m^2^

^e^ Abdominal obesity defined as waist circumference ≥94 cm for men and ≥80 cm for women

^f^ High BP defined as blood pressure ≥140/90 mmHg or reported treatment for high blood pressure

**^◊^** Also adjusted for BMI

* *P* <0.05

** *P* <0.001
