# Supplementary material for: Urbanicity and Lifestyle Risk Factors for Cardiometabolic Diseases in Rural Uganda: A Cross-Sectional Study
Source: PLoS Med. 2014 Jul 29;11(7):e1001683. doi: 10.1371/journal.pmed.1001683 (PMC4114555; doi:10.1371/journal.pmed.1001683)
Supplement: Table S5 — Unadjusted and adjusted associations between a 1–standard deviation change in urbanicity and lifestyle risk factors by sex, Uganda, 2011. (DOCX) [file pmed.1001683.s005.docx]

**Table S5. Unadjusted and adjusted associations between a 1-standard deviation change in urbanicity and lifestyle risk factors by sex, Uganda, 2011**

| Lifestyle Risk Factor |  | Unadjusted |  | Adjusted for age, sex and household clustering |  | Adjusted for age, sex SES, and household clustering |  | P-value (test for non-linearity) ^▲^ |
| --- | --- | --- | --- | --- | --- | --- | --- | --- |
|  |  | Coef. (95%CI) |  | Coef. (95%CI) |  | Coef. (95%CI) |  |  |
| Total^†^ |  |  |  |  |  |  |  |  |
| Alcohol intake (number of drinks per day) |  | 0.05* (0.02, 0.09) |  | 0.07** (0.03, 0.11) |  | 0.09** (0.05, 0.13) |  | 0.86 |
| Fruit and vegetable consumption (number per day) |  | -0.32** (-0.38, -0.25) |  | -0.32** (-0.39, -0.26) |  | -0.32** (-0.39, -0.26) |  | 0.14 |
| Physical activity (number of minutes per week) |  | -34.52* (-56.52, -12.52) |  | -33.98* (-58.11, -9.84) |  | -30.68* (-55.18, -6.17) |  | 0.02 |
| BMI |  | 0.32** (0.23, 0.41) |  | 0.38** (0.28, 0.48) |  | 0.31** (0.21, 0.40) |  | 0.06 |
| WC |  | 0.49** (0.28, 0.70) |  | 0.73** (0.51, 0.95) |  | 0.57** (0.35, 0.79) |  | 0.42 |
| SBP (mmHg) ^◊^ |  | -0.48* (-0.87, -0.09) |  | -0.12 (-0.45, 0.22) |  | -0.16 (-0.50, 0.18) |  | 0.72 |
| DBP (mmHg) ^◊^ |  | 0.03 (-0.21, 0.26) |  | 0.19 (-0.04, 0.41) |  | 0.16 (-0.07, 0.39) |  | 0.06 |
| Men |  |  |  |  |  |  |  |  |
| Alcohol intake (number of drinks per day) |  | 0.06* (0.01, 0.12) |  | 0.08* (0.02, 0.13) |  | 0.09* (0.03, 0.15) |  | - |
| Fruit and vegetable consumption (number per day) |  | -0.34** (-0.44, -0.23) |  | -0.36** (-0.45, -0.27) |  | -0.35** (-0.45, -0.26) |  | - |
| Physical activity (number of minutes per week) |  | -7.39 (-46.29, 31.52) |  | -7.67 (-49.63, 34.29) |  | -2.58 (-45.58, 40.43) |  | - |
| BMI |  | 0.27** (0.16, 0.38) |  | 0.31** (0.19, 0.43) |  | 0.25** (0.13, 0.37) |  | - |
| WC |  | 0.56** (0.29, 0.83) |  | 0.74** (0.46, 1.02) |  | 0.61** (0.33, 0.89) |  | - |
| SBP (mmHg) ^◊^ |  | 0.14 (-0.42, 0.70) |  | 0.19 (-0.28, 0.67) |  | 0.10 (-0.38, 0.58) |  | - |
| DBP (mmHg) ^◊^ |  | 0.20* (-0.16, 0.56) |  | 0.32 (-0.01, 0.67) |  | 0.29 (-0.05, 0.64) |  | - |
| Women |  |  |  |  |  |  |  |  |
| Alcohol intake (number of drinks per day) |  | 0.05* (0.01, 0.09) |  | 0.06* (0.01, 0.11) |  | 0.07* (0.02, 0.13) |  | - |
| Fruit and vegetable consumption (number per day) |  | -0.30** (-0.38, -0.21) |  | -0.30** (-0.39, -0.22) |  | -0.31** (-0.39, -0.23) |  | - |
| Physical activity (number of minutes per week) |  | -52.37** (-76.40, -28.33) |  | -52.48** (-79.53, -25.43) |  | -50.61** (-77.83, -23.38) |  | - |
| BMI |  | 0.35** (0.22, 0.48) |  | 0.40** (0.26, 0.54) |  | 0.32** (0.18, 0.46) |  | - |
| WC |  | 0.41* (0.11, 0.72) |  | 0.65* (0.33, 0.96) |  | 0.47* (0.15, 0.79) |  | - |
| SBP (mmHg) ^◊^ |  | -0.93* (-1.46, -0.40) |  | -0.36 (-0.81, 0.08) |  | -0.36 (-0.81, -0.09) |  | - |
| DBP (mmHg) ^◊^ |  | -0.11 (-0.42, 0.19) |  | 0.05 (-0.23, 0.34) |  | 0.04 (-0.25, 0.33) |  | - |

Abbreviations: BMI, body mass index; WC, waist circumference; SBP, systolic blood pressure; DBP, diastolic blood pressure; CI, confidence interval.

^†^ All estimates for the total population were also adjusted for sex.

**^◊^** Also adjusted for BMI

^▲^ Test for non-linearity based on fractional polynomials used to model associations considering urbanicity and outcomes as continuous data.

* *P* <0.05

** *P* <0.00
