# Supplementary material for: Urbanicity and Lifestyle Risk Factors for Cardiometabolic Diseases in Rural Uganda: A Cross-Sectional Study
Source: PLoS Med. 2014 Jul 29;11(7):e1001683. doi: 10.1371/journal.pmed.1001683 (PMC4114555; doi:10.1371/journal.pmed.1001683)
Supplement: Table S6 — Numbers and proportions of participants with missing data by urbanicity quartile, General Population Cohort, Uganda, 2011. (DOCX) [file pmed.1001683.s006.docx]

**Table S6. Numbers and proportions of participants with missing data by urbanicity quartile, General Population Cohort, Uganda, 2011**

|  | Urbanicity level | | | | | | |  |  |  |  |
| --- | --- | --- | --- | --- | --- | --- | --- | --- | --- | --- | --- |
| Variable | Quartile 1  (least urban)  (n=2,054)  n (%) |  | Quartile 2  (n=1,978)  n (%) |  | Quartile 3  (n=2,013)  n (%) |  | Quartile 4  (most urban)  (n=1,764)  n (%) |  | P-value◊ (difference between missing and urbanicity) |  | Proportion missing of total surveyed (n=7,809)  n (%) |
| No longer in the GPC dataset | - |  | - |  | - |  | - |  | - |  | 22 (0.3) |
| Age | 0 (0.0) |  | 0 (0.0) |  | 0 (0.0) |  | 0 (0.0) |  | - |  | 0 (0.0) |
| Sex | 0 (0.0) |  | 0 (0.0) |  | 0 (0.0) |  | 0 (0.0) |  | - |  | 0 (0.0) |
| Education | 0 (0.0) |  | 0 (0.0) |  | 0 (0.0) |  | 0 (0.0) |  | - |  | 0 (0.0) |
| Occupation those aged ≥18 years | 48 (3.1)▲ |  | 99 (6.7) ▲ |  | 106 (7.2) ▲ |  | 94 (6.8) ▲ |  | <0.001 |  | 347 (5.9) ▲ |
| SES | 176 (8.6) |  | 77 (3.9) |  | 87 (4.3) |  | 129 (7.3) |  | <0.001 |  | 468 (6.0) |
| Roof type | 21 (1.0) |  | 16 (0.8) |  | 19 (0.9) |  | 43 (2.4) |  | <0.001 |  | 99 (1.3) |
| Roof quality | 19 (0.9) |  | 16 (0.8) |  | 19 (0.9) |  | 41 (2.3) |  | <0.001 |  | 95 (1.2) |
| Wall type | 26 (1.3) |  | 18 (0.9) |  | 24 (1.2) |  | 44 (2.5) |  | <0.001 |  | 112 (1.4) |
| Ratio of rooms to people | 19 (0.9) |  | 16 (0.8) |  | 17 (0.8) |  | 41 (2.3) |  | <0.001 |  | 93 (1.2) |
| House ownership | 19 (0.9) |  | 16 (0.8) |  | 17 (0.8) |  | 41 (2.3) |  | <0.001 |  | 93 (1.2) |
| Own property for rental | 19 (0.9) |  | 16 (0.8) |  | 17 (0.8) |  | 41 (2.3) |  | <0.001 |  | 93 (1.2) |
| Ownership status of land | 19 (0.9) |  | 16 (0.8) |  | 17 (0.8) |  | 43 (2.4) |  | <0.001 |  | 95 (1.2) |
| Own other land | 19 (0.9) |  | 16 (0.8) |  | 18 (0.9) |  | 44 (2.5) |  | <0.001 |  | 97 (1.2) |
| Amount of other land owned | 167 (8.1) |  | 75 (3.8) |  | 78 (3.9) |  | 123 (6.9) |  | <0.001 |  | 443 (5.7) |
| Employment of workers | 21 (1.0) |  | 16 (0.8) |  | 17 (0.8) |  | 42 (2.4) |  | <0.001 |  | 96 (1.2) |
| Number of workers | 21 (1.0) |  | 16 (0.8) |  | 17 (0.8) |  | 42 (2.4) |  | <0.001 |  | 96 (1.2) |
| Smoking | 0 (0.00) |  | 0 (0.00) |  | 0 (0.0) |  | 0 (0.0) |  | - |  | 0 (0.0) |
| Alcohol consumption | 0 (0.0) |  | 0 (0.0) |  | 0 (0.0) |  | 0 (0.0) |  | - |  | 0 (0.0) |
| Fruit and vegetable consumption | 8 (0.4) |  | 6 (0.3) |  | 5 (0.2) |  | 7 (0.4) |  | 0.83^†^ |  | 26 (0.3) |
| Physical activity | 0 (0.0) |  | 0 (0.0) |  | 0 (0.0) |  | 1 (0.06) |  | 0.33^†^ |  | 1 (0.01) |
| BMI | 66 (3.2) |  | 69 (3.5) |  | 58 (2.9) |  | 63 (3.6) |  | 0.62 |  | 256 (3.3) |
| Waist circumference | 64 (3.1) |  | 71 (3.6) |  | 61 (3.0) |  | 61 (3.5) |  | 0.72 |  | 257 (3.3) |
| Blood pressure | 5 (0.2) |  | 3 (0.1) |  | 6 (0.3) |  | 2 (0.1) |  | 0.57^†^ |  | 16 (0.2) |

**▲** Data restricted to those aged **≥**18 years (quartile 1: n = 1,533, quartile 2: n = 1,483, quartile 3: n = 1,465, quartile 4: n = 1,375; overall: n = 5,856)

◊ Chi-square test used to compare the proportion of participants with missing data across urbanicity quartiles.

† Fisher’s exact test used to compare the proportion of participants with missing data across urbanicity quartiles.
