# Supplementary material for: Urbanicity and Lifestyle Risk Factors for Cardiometabolic Diseases in Rural Uganda: A Cross-Sectional Study
Source: PLoS Med. 2014 Jul 29;11(7):e1001683. doi: 10.1371/journal.pmed.1001683 (PMC4114555; doi:10.1371/journal.pmed.1001683)
Supplement: Table S7 — Characteristics and prevalence of lifestyle risk factors among those with and without socioeconomic status data overall and by urbanicity quartile, General Population Cohort, Uganda, 2011. (DOCX) [file pmed.1001683.s007.docx]

**Table S7. Characteristics and prevalence of lifestyle risk factors among those with and without socioeconomic status data overall and by urbanicity quartile, General Population Cohort, Uganda, 2011**

| Variable |  | Without SES data  n (%) |  | With SES data  n (%) |  | P-value (comparison of those with and without data) |
| --- | --- | --- | --- | --- | --- | --- |
| Overall |  | 469 (100.0) |  | 7,340 (100.0) |  | - |
| Mean age - years (SD) |  | 34.4 (18.5) |  | 34.3 (18.3) |  | 0.96 |
| Proportion of men |  | 202 (43.1) |  | 3,215 (43.8) |  | 0.76 |
| Ethnic origin - Ugandan |  | 310 (79.5) |  | 5,780 (79.7) |  | 0.92 |
| Educated past primary school |  | 132 (28.1) |  | 1,900 (25.9) |  | 0.28 |
| Employed |  | 295 (80.3) |  | 4,878 (90.3) |  | 0.23 |
| Current smokers |  | 38 (8.1) |  | 603 (8.2) |  | 0.93 |
| Heavy drinkers |  | 7 (1.5) |  | 58 (0.8) |  | 0.10 |
| Low fruit and vegetable consumption |  | 368 (78.6) |  | 5,5,83 (76.3) |  | 0.25 |
| Low physical activity |  | 290 (61.8) |  | 4,389 (59.8) |  | 0.38 |
| High BMI |  | 66 (14.7) |  | 868 (12.2) |  | 0.12 |
| Abdominal obesity |  | 87 (19.3) |  | 1,254 (17.6) |  | 0.34 |
| High blood pressure |  | 62 (13.2) |  | 1,203 (16.4) |  | 0.07 |
| Urbanicity quartile 1 (most rural) |  | 176 (100.0) |  | 1,878 (100.0) |  | - |
| Mean age - years (SD) |  | 37.5 (20.6) |  | 35.2 (18.9) |  | 0.13 |
| Proportion of men |  | 78 (44.3) |  | 820 (43.6) |  | 0.87 |
| Ethnic origin - Ugandan |  | 123 (76.4) |  | 1,427 (77.2) |  | 0.82 |
| Educated past primary school |  | 34 (19.3) |  | 317 (16.9) |  | 0.41 |
| Employed |  | 118 (90.1) |  | 1,297 (91.3) |  | 0.64 |
| Current smokers |  | 14 (7.9) |  | 170 (9.0) |  | 0.63 |
| Heavy drinkers |  | 2 (1.1) |  | 9 (0.5) |  | 0.25 |
| Low fruit and vegetable consumption |  | 121 (69.1) |  | 1,292 (69.0) |  | 0.98 |
| Low physical activity |  | 112 (63.6) |  | 1,050 (55.9) |  | 0.05 |
| High BMI |  | 26 (15.3) |  | 187 (10.3) |  | 0.04* |
| Abdominal obesity |  | 34 (19.9) |  | 295 (16.2) |  | 0.22 |
| High blood pressure |  | 32 (18.3) |  | 330 (17.6) |  | 0.82 |
| Urbanicity quartile 2 |  | 77 (100.0) |  | 1,901 (100.0) |  | - |
| Mean age - years (SD) |  | 31.5 (17.4) |  | 34.6 (18.6) |  | 0.15 |
| Proportion of men |  | 35 (45.4) |  | 824 (43.3) |  | 0.71 |
| Ethnic origin - Ugandan |  | 52 (82.5) |  | 1,457 (77.4) |  | 0.34 |
| Educated past primary school |  | 22 (28.6) |  | 410 (21.6) |  | 0.14 |
| Employed |  | 43 (84.3) |  | 1,239 (89.1) |  | 0.28 |
| Current smokers |  | 5 (6.5) |  | 154 (8.1) |  | 0.61 |
| Heavy drinkers |  | 2 (2.6) |  | 16 (0.8) |  | 0.11 |
| Low fruit and vegetable consumption |  | 60 (77.9) |  | 1,527 (80.6) |  | 0.56 |
| Low physical activity |  | 51 (66.2) |  | 1,133 (59.6) |  | 0.24 |
| High BMI |  | 8 (10.8) |  | 215 (11.7) |  | 0.81 |
| Abdominal obesity |  | 11 (14.7) |  | 346 (18.9) |  | 0.36 |
| High blood pressure |  | 8 (10.4) |  | 313 (16.5) |  | 0.15 |
| Urbanicity quartile 3 |  | 87 (100.0) |  | 1,926 (100.0) |  | - |
| Mean age - years (SD) |  | 32.8 (17.2) |  | 34.5 (18.7) |  | 0.40 |
| Proportion of men |  | 35 (40.2) |  | 859 (44.6) |  | 0.42 |
| Ethnic origin - Ugandan |  | 58 (77.3) |  | 1,561 (81.9) |  | 0.32 |
| Educated past primary school |  | 23 (26.4) |  | 502 (26.1) |  | 0.94 |
| Employed |  | 53 (86.9) |  | 1,236 (91.3) |  | 0.24 |
| Current smokers |  | 6 (6.9) |  | 162 (8.4) |  | 0.62 |
| Heavy drinkers |  | 1 (1.1) |  | 15 (0.8) |  | 0.70 |
| Low fruit and vegetable consumption |  | 73 (83.9) |  | 1,425 (74.1) |  | 0.04* |
| Low physical activity |  | 48 (55.2) |  | 1,132 (58.8) |  | 0.50 |
| High BMI |  | 18 (21.4) |  | 217 (11.6) |  | 0.007* |
| Abdominal obesity |  | 22 (26.2) |  | 309 (16.5) |  | 0.02* |
| High blood pressure |  | 10 (11.5) |  | 313 (16.3) |  | 0.23 |
| Urbanicity quartile 4 (least rural) |  | 129 (100.0) |  | 1,635 (100.0) |  | - |
| Mean age - years (SD) |  | 32.8 (16.6) |  | 32.7 (16.4) |  | 0.94 |
| Proportion of men |  | 54 (41.8) |  | 712 (43.5) |  | 0.71 |
| Ethnic origin - Ugandan |  | 77 (84.6) |  | 1,335 (82.6) |  | 0.63 |
| Educated past primary school |  | 53 (41.1) |  | 671 (41.0) |  | 0.99 |
| Employed |  | 81 (89.0) |  | 1,106 (89.5) |  | 0.89 |
| Current smokers |  | 13 (10.1) |  | 117 (7.2) |  | 0.22 |
| Heavy drinkers |  | 2 (1.5) |  | 18 (1.1) |  | 0.64 |
| Low fruit and vegetable consumption |  | 114 (88.4) |  | 1,339 (82.2) |  | 0.08 |
| Low physical activity |  | 79 (61.2) |  | 1,074 (65.7) |  | 0.30 |
| High BMI |  | 14 (11.6) |  | 249 (15.8) |  | 0.22 |
| Abdominal obesity |  | 20 (16.5) |  | 304 (19.2) |  | 0.47 |
| High blood pressure |  | 12 (9.3) |  | 247 (15.1) |  | 0.07 |
