# Supplementary material for: Urbanicity and Lifestyle Risk Factors for Cardiometabolic Diseases in Rural Uganda: A Cross-Sectional Study
Source: PLoS Med. 2014 Jul 29;11(7):e1001683. doi: 10.1371/journal.pmed.1001683 (PMC4114555; doi:10.1371/journal.pmed.1001683)
Supplement: Table S8 — Characteristics and prevalence of lifestyle risk factors among participants aged ≥18 y with and without primary occupation data overall and by urbanicity quartile, General Population Cohort, Uganda, 2011. (DOCX) [file pmed.1001683.s008.docx]

**Table S8. Characteristics and prevalence of lifestyle risk factors among participants aged ≥18 years with and without primary occupation data overall and by urbanicity quartile, General Population Cohort, Uganda, 2011**

| Variable | Without primary occupation data  n (%) |  | With primary occupation data  n (%) |  | P-value (comparison of those with and without data) | |  |
| --- | --- | --- | --- | --- | --- | --- | --- |
| Overall | 347 (100.0) |  | 5,509 (100.0) |  | - |  | |
| Mean age - years (SD) | 20.2 (2.3) |  | 41.9 (16.6) |  | <0.001 |  | |
| Proportion of men | 197 (56.8) |  | 2,219 (40.3) |  | <0.001 |  | |
| Ethnic origin - Ugandan | 288 (83.5) |  | 4,195 (78.1) |  | 0.019 |  | |
| Educated past primary school | 265 (76.4) |  | 1,339 (24.3) |  | <0.001 |  | |
| Current smokers | 1 (0.3) |  | 634 (11.5) |  | <0.001 |  | |
| Heavy drinkers | 0 (0.0) |  | 65 (1.2) |  | 0.042 |  | |
| Low fruit and vegetable consumption | 261 (75.6) |  | 4,215 (76.8) |  | 0.619 |  | |
| Low physical activity | 204 (58.8) |  | 3,269 (59.3) |  | 0.84 |  | |
| High BMI | 23 (6.6) |  | 865 (16.4) |  | <0.001 |  | |
| Abdominal obesity | 20 (5.8) |  | 1,263 (23.9) |  | <0.001 |  | |
| High blood pressure | 27 (7.8) |  | 1,141 (20.8) |  | <0.001 |  | |
| Urbanicity quartile 1 (most rural) | 48 (100.0) |  | 1,485 (100.0) |  | - |  | |
| Mean age - years (SD) | 19.7 (1.7) |  | 43.0 (17.2) |  | <0.001 |  | |
| Proportion of men | 25 (52.1) |  | 607 (40.1) |  | 0.121 |  | |
| Ethnic origin - Ugandan | 40 (83.3) |  | 1,088 (75.1) |  | 0.192 |  | |
| Educated past primary school | 37 (77.1) |  | 244 (16.4) |  | <0.001 |  | |
| Current smokers | 0 (0.0) |  | 182 (12.3) |  | <0.001 |  | |
| Heavy drinkers | 0 (0.0) |  | 11 (0.7) |  | 0.550 |  | |
| Low fruit and vegetable consumption | 32 (66.7) |  | 1,028 (69.5) |  | 0.669 |  | |
| Low physical activity | 25 (52.1) |  | 840 (56.6) |  | 0.538 |  | |
| High BMI | 1 (2.1) |  | 203 (14.3) |  | 0.018 |  | |
| Abdominal obesity | 1 (2.1) |  | 313 (21.9) |  | 0.001 |  | |
| High blood pressure | 7 (14.6) |  | 328 (22.2) |  | 0.212 |  | |
| Urbanicity quartile 2 | 99 (100.0) |  | 1,384 (100.0) |  | - |  | |
| Mean age - years (SD) | 20.2 (2.1) |  | 42.4 (16.7) |  | <0.001 |  | |
| Proportion of men | 52 (52.5) |  | 551 (39.8) |  | 0.013 |  | |
| Ethnic origin - Ugandan | 81 (81.8) |  | 1,034 (76.1) |  | 0.198 |  | |
| Educated past primary school | 76 (76.8) |  | 261 (18.9) |  | <0.001 |  | |
| Current smokers | 0 (0.0) |  | 158 (11.4) |  | <0.001 |  | |
| Heavy drinkers | 0 (0.0) |  | 18 (1.3) |  | 0.254 |  | |
| Low fruit and vegetable consumption | 79 (79.8) |  | 1,105 (80.2) |  | 0.925 |  | |
| Low physical activity | 59 (59.6) |  | 818 (59.1) |  | 0.923 |  | |
| High BMI | 8 (8.1) |  | 201 (15.3) |  | 0.053 |  | |
| Abdominal obesity | 6 (6.1) |  | 340 (25.8) |  | <0.001 |  | |
| High blood pressure | 9 (9.1) |  | 286 (20.7) |  | 0.005 |  | |
| Urbanicity quartile 3 | 106 (100.0) |  | 1,359 (100.0) |  | - |  | |
| Mean age - years (SD) | 20.4 (3.00) |  | 43.3 (16.5) |  | <0.001 |  | |
| Proportion of men | 62 (58.4) |  | 545 (40.1) |  | <0.001 |  | |
| Ethnic origin - Ugandan | 89 (85.6) |  | 1,067 (79.9) |  | 0.163 |  | |
| Educated past primary school | 84 (79.2) |  | 301 (22.1) |  | <0.001 |  | |
| Current smokers | 1 (0.9) |  | 166 (12.2) |  | <0.001 |  | |
| Heavy drinkers | 0 (0.0) |  | 16 (1.2) |  | 0.261 |  | |
| Low fruit and vegetable consumption | 75 (70.7) |  | 1,024 (75.6) |  | 0.263 |  | |
| Low physical activity | 56 (52.8) |  | 796 (58.6) |  | 0.248 |  | |
| High BMI | 7 (6.6) |  | 216 (16.5) |  | 0.007 |  | |
| Abdominal obesity | 8 (7.5) |  | 307 (23.5) |  | <0.001 |  | |
| High blood pressure | 5 (4.7) |  | 291 (21.5) |  | <0.001 |  | |
| Urbanicity quartile 4 (least rural) | 94 (100.0) |  | 1,281 (100.0) |  | - |  | |
| Mean age - years (SD) | 20.1 (1.8) |  | 38.8 (15.2) |  | <0.001 |  | |
| Proportion of men | 58 (61.7) |  | 516 (40.3) |  | <0.001 |  | |
| Ethnic origin - Ugandan | 78 (82.9) |  | 1,006 (81.9) |  | 0.785 |  | |
| Educated past primary school | 68 (72.3) |  | 533 (41.6) |  | <0.001 |  | |
| Current smokers | 0 (0.0) |  | 128 (9.9) |  | 0.001 |  | |
| Heavy drinkers | 0 (0.0) |  | 20 (1.6) |  | 0.222 |  | |
| Low fruit and vegetable consumption | 75 (81.5) |  | 1,058 (82.8) |  | 0.745 |  | |
| Low physical activity | 64 (68.1) |  | 815 (63.7) |  | 0.390 |  | |
| High BMI | 7 (7.4) |  | 245 (20.1) |  | 0.003 |  | |
| Abdominal obesity | 5 (5.3) |  | 303 (24.8) |  | <0.001 |  | |
| High blood pressure | 6 (6.4) |  | 236 (18.4) |  | 0.003 |  | |
