# Supplementary material for: Urbanicity and Lifestyle Risk Factors for Cardiometabolic Diseases in Rural Uganda: A Cross-Sectional Study
Source: PLoS Med. 2014 Jul 29;11(7):e1001683. doi: 10.1371/journal.pmed.1001683 (PMC4114555; doi:10.1371/journal.pmed.1001683)
Supplement: Table S9 — Associations between increasing urbanicity and lifestyle risk factors adjusted for age, socioeconomic status, and clustering at household level, assuming those with missing primary occupation data are all involved or not involved in agriculture, General Population Cohort, Uganda, 2011. (DOCX) [file pmed.1001683.s009.docx]

**Table S9. Associations between increasing urbanicity and lifestyle risk factors adjusted for age, socioeconomic status, and clustering at household level, assuming those with missing primary occupation data are all involved or not involved in agriculture, General Population Cohort, Uganda, 2011**

| Lifestyle Risk Factor | Urbanicity level | | | | | | |
| --- | --- | --- | --- | --- | --- | --- | --- |
|  | Quartile 1 (least urban) |  | Quartile 2 |  | Quartile 3 |  | Quartile 4 (most urban) |
|  | RR |  | Difference in mean (95%CI) |  | Difference in mean (95%CI) |  | Difference in mean (95%CI) |
| Involved in agriculture ^†^ |  |  |  |  |  |  |  |
| Current smokers | 1 |  | 0.96 (0.78, 1.17) |  | 0.96 (0.79, 1.16) |  | 1.10 (0.89, 1.37) |
| Heavy drinkers ^a^ | 1 |  | 1.92 (0.82, 4.46) |  | 1.67 (0.71, 3.89) |  | 3.34* (1.43, 7.82) |
| Low fruit and vegetable consumption ^b^ | 1 |  | 1.17** (1.12, 1.22) |  | 1.07* (1.02, 1.13) |  | 1.19** (1.14, 1.24) |
| Low physical activity ^c^ | 1 |  | 1.06* (1.00, 1.13) |  | 1.05 (0.99, 1.11) |  | 1.17** (1.10, 1.23) |
| High BMI ^d^ | 1 |  | 1.14 (0.95, 1.37) |  | 1.13 (0.93, 1.37) |  | 1.48** (1.24, 1.77) |
| Abdominal obesity ^e^ | 1 |  | 1.17* (1.02, 1.33) |  | 1.05 (0.91, 1.21) |  | 1.21* (1.05, 1.38) |
| High BP ^f◊^ | 1 |  | 0.95 (0.83, 1.08) |  | 0.93 (0.81, 1.06) |  | 0.97 (0.84, 1.12) |
| Not involved in agriculture ^▲^ |  |  |  |  |  |  |  |
| Current smokers | 1 |  | 0.96 (0.78, 1.17) |  | 0.96 (0.79, 1.16) |  | 1.10 (0.88, 1.37) |
| Heavy drinkers ^a^ | 1 |  | 1.92 (0.82, 4.46) |  | 1.67 (0.71, 3.89) |  | 3.34* (1.43, 7.82) |
| Low fruit and vegetable consumption ^b^ | 1 |  | 1.17** (1.12, 1.22) |  | 1.07* (1.02, 1.13) |  | 1.19** (1.14, 1.24) |
| Low physical activity ^c^ | 1 |  | 1.06* (1.00, 1.13) |  | 1.05 (0.99, 1.11) |  | 1.17** (1.10, 1.23) |
| High BMI ^d^ | 1 |  | 1.14 (0.95, 1.37) |  | 1.13 (0.93, 1.37) |  | 1.48** (1.24, 1.77) |
| Abdominal obesity ^e^ | 1 |  | 1.17* (1.02, 1.33) |  | 1.05 (0.91, 1.21) |  | 1.21* (1.05, 1.38) |
| High BP ^f◊^ | 1 |  | 0.95 (0.83, 1.08) |  | 0.93 (0.81, 1.06) |  | 0.97 (0.84, 1.12) |

Abbreviations: BMI, body mass index; BP, blood pressure; CI, confidence interval; RR, risk ratio.

**^†^** All associations are based on urbanicity scores recalculated assuming all adults with missing primary occupation data are involved in agriculture as their primary occupation.

^a^ Heavy drinkers defined as any woman who reports drinking more than one drink per day or any man who reports drinking more than two drinks per day.

^b^ Low fruit and vegetable consumption defined as eating less than five portions of fruit or vegetables per day

^c^ Low physical activity defined as doing less than 5 days a week of any combination of walking, moderate or vigorous intensity activities and less than 600 minutes of physical activity per week

^d^ High BMI defined as BMI ≥ 25kg/m^2^

^e^ Abdominal obesity defined as waist circumference ≥94 cm for men and ≥80 cm for women

^f^ High BP defined as blood pressure ≥140/90 mmHg or reported treatment for high blood pressure

**^◊^** Also adjusted for BMI

**^▲^** All associations are based on urbanicity scores recalculated assuming all adults with missing primary occupation data are not involved in agriculture as their primary occupation.

* *P* <0.05

** *P* <0.001
