# Supplementary material for: Urbanicity and Lifestyle Risk Factors for Cardiometabolic Diseases in Rural Uganda: A Cross-Sectional Study
Source: PLoS Med. 2014 Jul 29;11(7):e1001683. doi: 10.1371/journal.pmed.1001683 (PMC4114555; doi:10.1371/journal.pmed.1001683)
Supplement: Table S10 — Associations between a 1–standard deviation change in urbanicity and lifestyle risk factors adjusted for age, socioeconomic status, and clustering at household level, assuming those with missing primary occupation data are all involved or not involved in agriculture, General Population Cohort, Uganda, 2011. (DOCX) [file pmed.1001683.s010.docx]

**Table S10. Associations between a 1-standard deviation change in urbanicity and lifestyle risk factors adjusted for age, socioeconomic status, and clustering at household level, assuming those with missing primary occupation data are all involved or not involved in agriculture, General Population Cohort, Uganda, 2011**

|  | Adjusted for age, sex SES, and household clustering |
| --- | --- |
|  | Coef. (95%CI) |
| Involved in agriculture ^†^ |  |
| Alcohol intake (number of drinks per day) | 0.09** (0.05, 0.13) |
| Fruit and vegetable consumption (number per day) | -0.33** (-0.39, -0.26) |
| Physical activity (number of minutes per week) | -30.52* (-55.18, -5.89) |
| BMI | 0.31** (0.21, 0.40) |
| WC | 0.57** (0.35, 0.79) |
| SBP (mmHg) ^◊^ | -0.15 (-0.49, 0.19) |
| DBP (mmHg) ^◊^ | 0.16 (-0.06, 0.40) |
| Not involved in agriculture ^▲^ |  |
| Alcohol intake (number of drinks per day) | 0.09** (0.05, 0.13) |
| Fruit and vegetable consumption (number per day) | -0.33** (-0.39, -0.26) |
| Physical activity (number of minutes per week) | -32.41* (-57.06, -7.75) |
| BMI | 0.31** (0.21, 0.41) |
| WC | 0.58** (0.35, 0.80) |
| SBP (mmHg) ^◊^ | -0.17 (-0.51, 0.17) |
| DBP (mmHg) ^◊^ | 0.15 (-0.08, 0.38) |

Abbreviations: BMI, body mass index; WC, waist circumference; SBP, systolic blood pressure; DBP, diastolic blood pressure; CI, confidence interval.

**^†^** All associations are based on urbanicity scores recalculated assuming all adults with missing primary occupation data are involved in agriculture as their primary occupation.

**^◊^** Also adjusted for BMI

**^▲^** All associations are based on urbanicity scores recalculated assuming all adults with missing primary occupation data are not involved in agriculture as their primary occupation.

* *P* <0.05

** *P* <0.00
